# Application of hot water and cold air to reduce bacterial contamination on broiler carcasses

**DOI:** 10.3389/fmicb.2024.1429756

**Published:** 2024-09-19

**Authors:** Anja Beterams, Alina Kirse, Lothar Kreienbrock, Kerstin Stingl, Niels Bandick, Felix Reich

**Affiliations:** ^1^Department of Biological Safety, German Federal Institute for Risk Assessment, Berlin, Germany; ^2^Institute of Biometry, Epidemiology and Information Processing (IBEI), WHO Collaborating Centre for Research and Training for Health at the Human-Animal-Environment Interface, University of Veterinary Medicine Hannover, Hanover, Germany

**Keywords:** broiler slaughtering process, thermal decontamination methods, *Campylobacter* spp., *Salmonella* spp., hygiene indicators, PMA qPCR, sensory analysis

## Abstract

Two physical treatments (heat via water bath and cold air) with various temperatures (20/70/75/80°C and − 80/−90°C) and exposure times (20, 30, 40 s) were carried out to identify a decontaminating effect on zoonotic pathogens on broiler carcasses. Subsequently, carcasses were analyzed for thermotolerant *Campylobacter (C.)*, *Salmonella*, *Escherichia (E.). coli* and total colony count (TCC). Moreover, for the hot water treatment, qPCR with viable/dead differentiation (v-qPCR) was applied to detect viable but non-culturable cells (VBNC) of *Campylobacter,* referred to as intact but putatively infectious units (IPIU). Hot water immersion was tested on carcasses inoculated with *C. jejuni* and *Salmonella*, while cold air treatment was evaluated for naturally contaminated carcasses of broiler flocks colonized with *Campylobacter*. For hot water treatment, the statistically significant reducing effect was about 1 log_10_ CFU/ml for both *Salmonella* and *Campylobacter* for 70–80°C and 20/30 s treatments. The effect of heat treatment for *Campylobacter* was smaller when samples were analyzed with v-qPCR with reductions of 0.5–0.8 log_10_ IPIU/ml in mean. Cold air treatments at −90°C were effective in reducing the mean contamination level of *Campylobacter* by 0.4–0.5 log_10_ CFU/ml at all exposure times (*p* < 0.05). Hot water treatments showed a decreasing trend on TCC by 0.6–0.9 log_10_ CFU/ml (p < 0.05). TCC counts were not significantly affected by cold air treatment. For *E. coli* no statistically significant reductions were observed by hot water treatment. The cold air treatment at −90°C for 20 and 40 s led to a reduction of *E. coli* by 0.4 and 0.8 log_10_ CFU/ml (*p* < 0.05), respectively. Treatment of carcasses with higher bacterial levels tended to show higher reduction. The research demonstrated that the efficacy of physical treatments for decontamination of broiler carcasses was more pronounced for hot water immersion than for cold air exposure. In conclusion, the results shed light on the potential application of these physical treatments in practice to reduce the quantitative load of contaminating pathogens to enhance food safety in the broiler meat production.

## 1 Introduction

Campylobacteriosis and salmonellosis are still the most important bacterial zoonoses transmitted via food. Broiler meat is of particular importance as it is one of the main reservoirs for *Campylobacter* and *Salmonella* as reported by the European Food Safety Authority (EFSA, 2021). Therefore, measures are needed for the broiler meat production to quantitatively reduce *Campylobacter* and *Salmonella* contamination in order to lower the number of human infections (EFSA, 2011).

Thermal processes such as heat or cold can reduce bacterial contamination of carcasses or meat. Scalding and chilling are thermal processes already implemented in the slaughtering process for technological purposes, which in themselves result in a reduction of bacteria on the meat (Berrang et al., 2011; Duffy et al., 2014). Heat is used in poultry slaughtering in the form of hot water immersion or steam when scalding carcasses (soft scalding: 51–53°C, 120–229 s; hard scalding: 55–58°C, 80–150 s) to loosen the feathers and allow for efficient plucking of carcasses (Dogan et al., 2022). This treatment can also affect microbiological contamination by washing excess dirt from carcasses and/or providing mild thermal inactivation of bacteria (Dogan et al., 2022). Chilling can either be applied by dry air or through immersion (Huezo et al., 2007). Thus, the slaughter process itself has the potential to reduce the initial bacterial load of carcasses, which was recently shown for European slaughterhouses (Beterams et al., 2024; Hauge et al., 2023; Hutchison et al., 2022). However, currently established measures are reaching their limits. To further improve hygiene, additional approaches should be considered. Recontamination of broiler carcasses frequently occurs after evisceration, which showed to be a crucial step within the slaughtering process (Beterams et al., 2024; Pacholewicz et al., 2015). In the beginning of the 2000s, several studies have been published on additional hot water treatment processes that can be used to reduce *Campylobacter* by around 1 log_10_ CFU on carcasses (Corry et al., 2007; Purnell et al., 2004; Whyte et al., 2003). For *Salmonella*, there had been only few studies, that focused on hot water immersion as an additional treatment, whereby hard scalding (McKee et al., 2008) and steam pasteurization (Kure et al., 2020) were the most relevant. However, the effect of hot water immersion on carcass contamination has been the subject in one recent study (Hauge et al., 2023).

Additional cold treatment beyond the standard cooling process can be used to reduce the bacterial load of *Campylobacter* by 0.5–1.5 log_10_ CFU/g (Haughton et al., 2012). Especially short-term treatments with ultra-cold air, leading to a superficial freezing of carcasses without changing the meat texture, showed promising reduction of *Campylobacter* of around 0.5 log_10_ CFU/carcass in pilot studies (Boysen and Rosenquist, 2009). New technologies in form of indirect liquid nitrogen cooling (SafeChill™) are implemented in the United Kingdom (Herrmann et al., 2017), but are yet not further evaluated in other countries and could provide new opportunities for decontamination of carcasses.

The microbial reduction of *Campylobacter* through additional treatments could be beneficial to improve the microbiological hygiene of poultry products. The effectiveness of *Campylobacter* intervention at slaughterhouse level has previously been studied with classical culturing methods (Boysen and Rosenquist, 2009; Corry et al., 2007; Haughton et al., 2012). To verify, if these methods reliably detect living cells, data generation can nowadays be complemented by molecular biological methods. Staining cells with propidium monoazide (PMA) before analysis with qPCR allows to differentiate live and potentially infectious *Campylobacter* cells from dead cells. Since this new PMA-dependent viable qPCR (v-qPCR) protocol including an internal sample process control had been validated for chicken rinse (Stingl et al., 2021), the technique can be applied for different settings and extended to other sample types and used to evaluate possible decontamination effects.

The aim of this study was to evaluate, whether physical treatment as an additional step post-evisceration can effectively reduce viable bacterial contamination on broiler carcasses. In particular, hot water immersion at 70, 75 and 80°C for 20 and 30 s and cold air treatment with the SafeChill™ technology at −80 and − 90°C for 20, 30 and 40 s were explored. The recent hot water treatment study was based on a specific re-contamination scenario, that was highlighted within a recent study on contamination in German broiler slaughterhouses (Beterams et al., 2024). Evisceration was identified as a relevant processing step, where the numbers of *Campylobacter* could increase and decontamination might contribute to improved meat hygiene. The hot water treatment was investigated with two quantification methods, microbiological and by v-qPCR. Lastly, it was investigated how both treatments would affect microbiological hygiene parameters for carcass contamination in poultry slaughtering, i.e., total colony count (TCC) and *Escherichia (E.) coli* concentrations.

## 2 Materials and methods

### 2.1 Hot water treatment trials

The treatment with hot water was conducted in a pilot scale plant at the German Federal Institute for Risk Assessment (BfR) in Berlin, Germany. For this process, a scalding tank was used, which worked with indirect heating of the water through a sheath filled with oil. The hot water treatment chosen here should be evaluated as a measure for decontamination after the evisceration step. Therefore, the carcasses used were collected at a broiler slaughterhouse after evisceration and neck removal to allow water to drain from the body cavity after treatment. To simulate fecal re-contamination during evisceration, the carcasses were inoculated with a combined suspension of *Campylobacter* and *Salmonella* strains (3 *Campylobacter jejuni* strains, one strain each of *S.* Enteritidis, *S.* Typhimurium, *S.* Infantis), to achieve a concentration of 4–5 log_10_ CFU/ml of whole carcass rinse (WCR), which was in the upper end of the range of carcass contamination after evisceration for *Campylobacter* (Beterams et al., 2024) and a similar concentration was chosen for *Salmonella*. Strains were provided and characterized by the National Reference Laboratories (NRLs) at the BfR and were isolated from broiler meat.

The bacteria were grown on Columbia agar with 5% sheep blood (ColB, OXOID, Wesel, Germany) for 24 h under microaerobic conditions at 41.5°C for *Campylobacter* and under aerobic conditions at 37°C for *Salmonella*. Cultures were adjusted to an OD_600_ of 0.05 in brain heart infusion broth (BHI, Article-Nr.: 1.10493.0500, Merck, Darmstadt, Germany) for *Campylobacter* and 0.2 in peptone water (PW, Article-Nr.: CM1049B, Thermo Fisher, Wesel, Germany) for *Salmonella* and incubated overnight to achieve a concentration of 8–9 log_10_ CFU/ml. The liquid cultures of three strains per genus were combined in equal amounts. For each strain 2 mL of the mixed suspension were diluted 1:2 with 0.85% NaCl/0.1% peptone water (1.12535, Merck, Darmstadt, Germany; LP0011 OXOID, Wesel, Germany) and then mixed 1:2 with cecal contents, that were free of *Campylobacter*, *Salmonella* and also *E. coli*. The concentrations of *Campylobacter* and *Salmonella* in this ready-to-use suspension were around 8 log_10_ CFU/ml, respectively. Broiler carcasses were inoculated with 0.1 mL of the suspension applied in the peri cloacal area and was incubated for 10 min at room temperature and normal atmosphere to allow bacteria to attach. Broilers were then treated with hot water, either at 70°C (± 2°C) for 30 s, at 75°C (± 2°C) or at 80°C (± 3°C) for 20 and 30 s, respectively. One control group without treatment and one group treated with water at room temperature (approximately 20°C) for 30 s were analyzed to evaluate the rinsing effect in itself. Broilers were sampled with WCR. The sample size was n = 17 per group and each setting was analyzed three times.

### 2.2 Cold treatment trials

**Figure 1:**
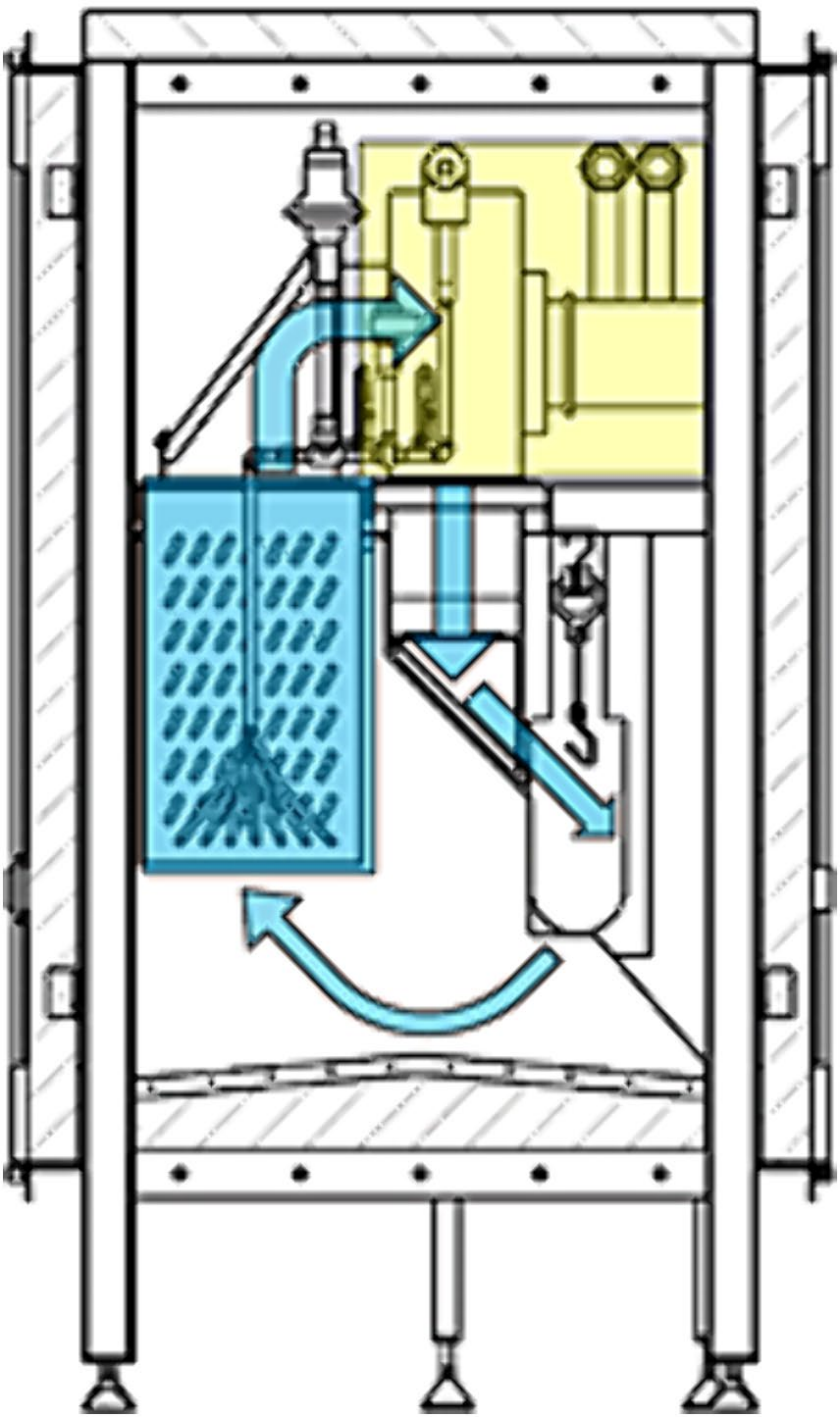
Workflow of the system; normal operation Freshline® SafeChill™ from Air Products; the adjustable system, where treatment time, which is controlled through line speed (20 to 60 s), air velocity (m/s) and the temperature (−70 to −110°C) can be changed, uses liquid nitrogen to cool process air by means of a heat exchanger. A fan circulates super chilled air, which is directed at the back of the eviscerated carcasses. The air is then re-circulated and cooled down again.

For the cold air treatment, the Freshline® SafeChill™ trial setup, provided by Air Products PLC (Figure 1), was placed inside the cooling area of a commercial broiler slaughterhouse, therefore only naturally contaminated carcasses could be used for the experiments. This system is meant as an additional processing step that can be integrated into the production line. It is an adjustable system, where treatment (cold exposure) time (min. 20 to max. 60 s), which is controlled by line speed, air velocity and the temperature (−70 to −110°C) can be changed. Liquid nitrogen is used to cool process air by means of a heat exchanger. A fan circulates super chilled air, which is directed at the back of the eviscerated carcasses. The air is then re-circulated and cooled down again.

The cold air treatment was conducted in two parts: In the first, four groups each were analyzed in parallel, i.e., three treatment times (20, 30, and 40 s) and a control group without treatment. Those treatment times were investigated at two different temperatures each (−80 or − 90 ± 2°C). Broilers were sampled with WCR. Due to technical issues, only two trials could be realized for −90°C 40 s in the first part of the study. In the second part, WCR and neck skin samples were analyzed in parallel in trials at −90°C for 30 s. For both parts and each treatment and control group 17 samples were collected per trial and every setting was repeated three times.

### 2.3 Microbiological analysis

In total, 1,122 samples were analyzed. Broiler carcasses were sampled with WCR by placing the carcass into a sterile bag (400×500 mm, PA/PE 90, sealed bag, HEIFO GmbH & Co. KG, Osnabrück, Germany), covered with 400 mL of 0.85% NaCl/0.1% peptone water (Article-Nr.: 1.12535, Merck., Darmstadt, Germany; LP0011 OXOID, Wesel, Germany). The carcasses were rinsed for 60 s by turning the bag 180° every 5 seconds and moving it vigorously up and down six to 10 times to allow rinsing fluid to also enter the body cavity so that all surfaces were rinsed. Around 200 mL of the WCR was filled into sterile plastic cups (300 mL, PP, VWR International GmbH, Darmstadt, Germany).

For the neck skin samples, around 10 g of skin were cut from the carcass by using a sterile knife and placing the sample into a 1 L plastic bag (freeze bag, ja!, REWE, Köln, Germany). All samples were transported to the laboratory refrigerated, stored under chilled conditions and analyzed within 24 h.

Individual neck skin samples were diluted ten-fold to achieve the first dilution, WCR were taken without further dilution directly. Initial and further ten-fold dilutions were prepared with 0.85% NaCl/0.1% peptone water. The total colony count (TCC) of the samples was determined by pipetting 1 mL of each tenfold dilution into petri dishes, poured over with plate-count agar (PCA, MAST Diagnostika GmbH, Reinfeld, Germany) and aerobically incubated at 30°C for 72 h based on ISO 4833-1:2014. *Escherichia coli* were enumerated with tryptone-bile-X-glucuronide agar (TBX, OXOID) using 1 mL of each ten-fold dilution and pour plating with aerobic incubation for 18–24 h at 44°C based on ISO 16649-2:2020. *Salmonella* spp. quantification was done by adapting standard plate count technique with selective agar and incubation conditions referring to ISO 6579-1:2017, by plating 0.1 mL of the respective dilution on xylose-lysine-deoxycholate agar (XLD, Merck) incubated aerobically at 37°C for 24 h. Thermophilic *Campylobacter* spp., were enumerated on modified charcoal cefoperazone deoxycholate agar (mCCDA, Merck/OXOID) with microaerobic incubation at 41.5°C for 48 h referring to ISO 10272-2:2017. To lower the detection limit for *Campylobacter* to 1 CFU/mL of WCR and 10 CFU/g of neck skin, 1 mL was plated onto three agar plates. The detection limit for *Salmonella* was 10 CFU/mL in WCR and for *E. coli* and TCC 1 CFU/mL in WCR or 10 CFU/g in neck skin samples.

### 2.4 Molecular biological analysis

The molecular biological quantification was performed for the samples of the hot water treatment. The protocols for *Campylobacter* were used according to Pacholewicz et al. (2019) and Stingl et al. (2021). Four 1 mL aliquots per sample were prepared in 1.5 mL reaction tubes (Eppendorf, Hamburg, Germany). Primers and probes, as well as the mastermix and DNA standards were used as described in Stingl et al. (2021). Two independent duplex qPCR protocols were used for the quantification of either thermotolerant *Campylobacter* spp. or the ISPC, each in combination with an internal amplification control (IPC-ntb2, (Anderson et al., 2011)). Standard curves were created for each run individually by comparing ct-values with the known content of genome copies of a standard series. A detailed protocol with spread sheet and equations can be found in the Supplementary materials in Stingl et al. (2021). An example of a standard curve and equation can be found in the Supplementary Figure S1. For every sample analysis, two aliquots were treated with PMA and two aliquots were analyzed without. The qPCR was performed with the ABI 7500 Real Time PCR System or the QuantStudio5 (Applied Biosystems by Thermo Fisher Scientific, Waltham, Massachusetts, United States), the cycling program was run as follows: 3 min at 95°C for initial denaturation; after that, 45 cycles with each 15 s at 95°C, 60 s at 60°C to measure the fluorescence and 30 s at 72°C.

Eight samples per group were analyzed by v-qPCR and compared with the results of the cultural analysis.

### 2.5 Sensory analysis

To identify the impact of hot water and cold air treatment on sensory characteristics of treated and untreated broiler carcasses the temperature under the skin was recorded (Datalogger Thermometer, HH520, Omega Engineering GmbH, Deckenpfronn, Germany) with a thermocouple type-T (5SC-TT-TI-30-1 M, Omega) inserted underneath the skin directly on top of the meat either on the center of the left breast muscle or the right upper leg and fixed with a string. Color was measured based on the CIELAB-system with a MINOLTA CR-210 (MINOLTA Camera Co., Ltd., Japan) device on the center of the left breast skin, as well as on the central part of the left thigh skin. The equipment was calibrated before measurements with a white standard tile and the lightness L*, a*(+) for red and b*(+) for yellow were measured. The visual appearance of carcasses was assessed based on pictures that were taken with a Nikon D3s camera (Macro lens Nikon 1:2.8 / *f* = 60 mm, sensor sensitivity = ISO 200, f-stop 32, 1/10 s; samples were illuminated with a Kaiser repro column with high-frequency/flare-free daylight (incident and transmitted light, Nikon Germany, Düsseldorf, Germany). The skin texture was described and assessed after visual analysis and palpation by two examiners with experience in poultry meat processing by comparing the samples in pairs of treated and untreated carcasses. In total, three carcasses per setting were analyzed before and after treatment, as well as after chilling for each group.

### 2.6 Statistical analysis

Data was transformed using the logarithm to the basis of 10 to normalize the data. In order to display the change in log_10_ CFU/ml, boxplots for each temperature and exposure time were created and compared to the control group. To examine the mean differences between the test and control group for the different temperature settings and exposure times for the hot water and cold air treatments, analysis of variance (ANOVA) and *post hoc*-tests were performed using Tukey’s test for equal sample sizes between the groups and Tukey–Kramer test for unequal sample sizes. The analyses where additionally supported by normal distributed residuals within the model calculations.

**Figure 2:**
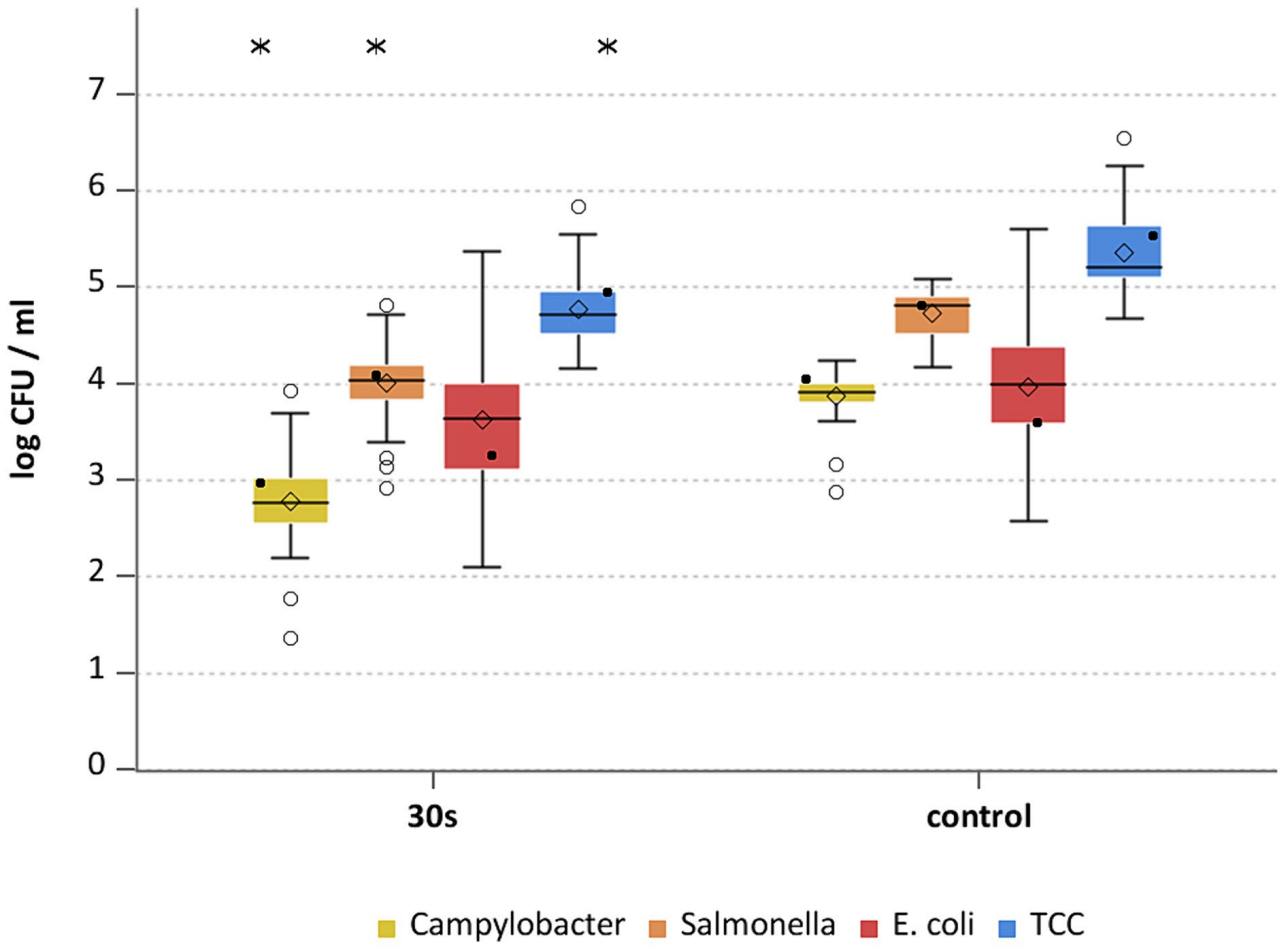
*Campylobacter* spp., *Salmonella* spp., *E. coli* and TCC levels of broiler carcasses after hot water immersion at 70°C for 30 s and corresponding untreated control group. Within box plots show the median (^___^), the least square mean (•), mean (◊) and outliers (o) are marked separately; * indicate statistically significant difference between treated groups and control group (*p* < 0.05, Tukey), (*n* = 51).

**Figure 3:**
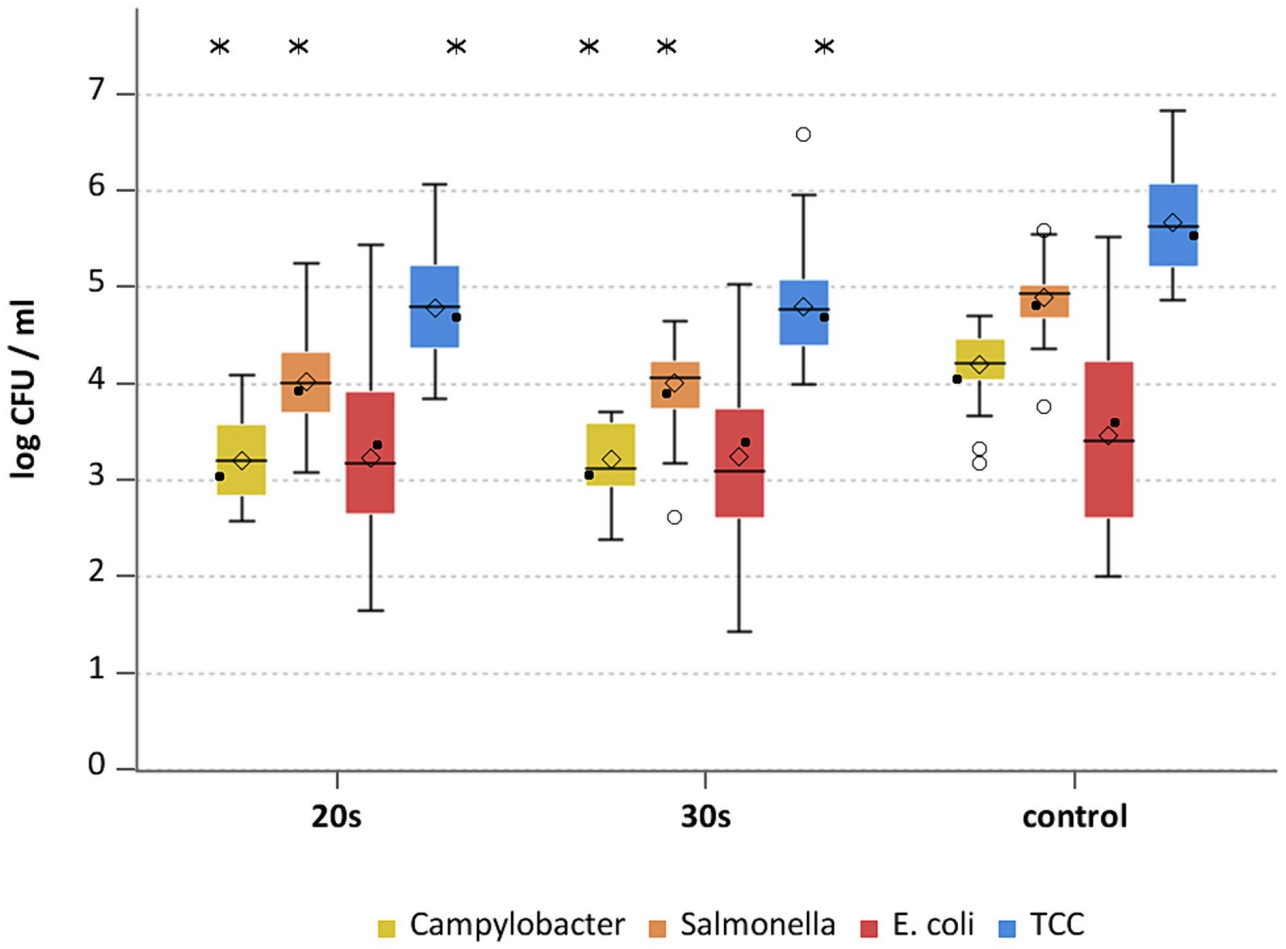
*Campylobacter* spp., *Salmonella* spp., *E. coli* and TCC levels of broiler carcasses after hot water immersion at 75°C for 20 and 30 s and corresponding untreated control group. Within box plots show the median (^___^), the least square mean (•), mean (◊) and outliers (o) are marked separately; * indicate statistically significant difference between treated groups and control group (*p* < 0.05, Tukey), (*n* = 51).

**Figure 4:**
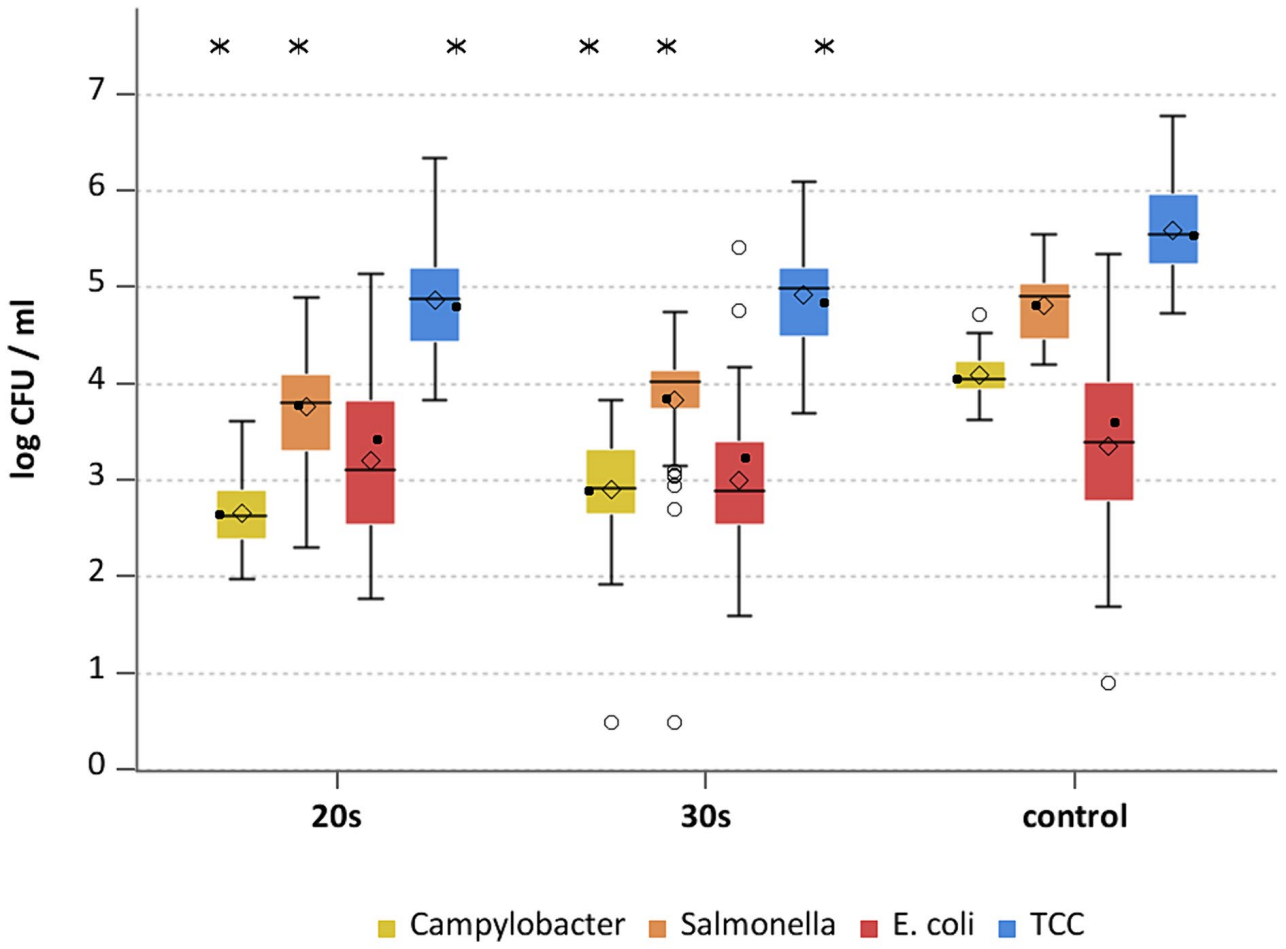
*Campylobacter* spp., *Salmonella* spp., *E. coli* and TCC levels of broiler carcasses after hot water immersion at 80°C for 20 and 30 s and corresponding untreated control group. Within box plots show the median (^___^), the least square mean (•), mean (◊) and outliers (o) are marked separately; * indicate statistically significant difference between treated groups and control group (*p* < 0.05, Tukey), (*n* = 51).

**Figure 5:**
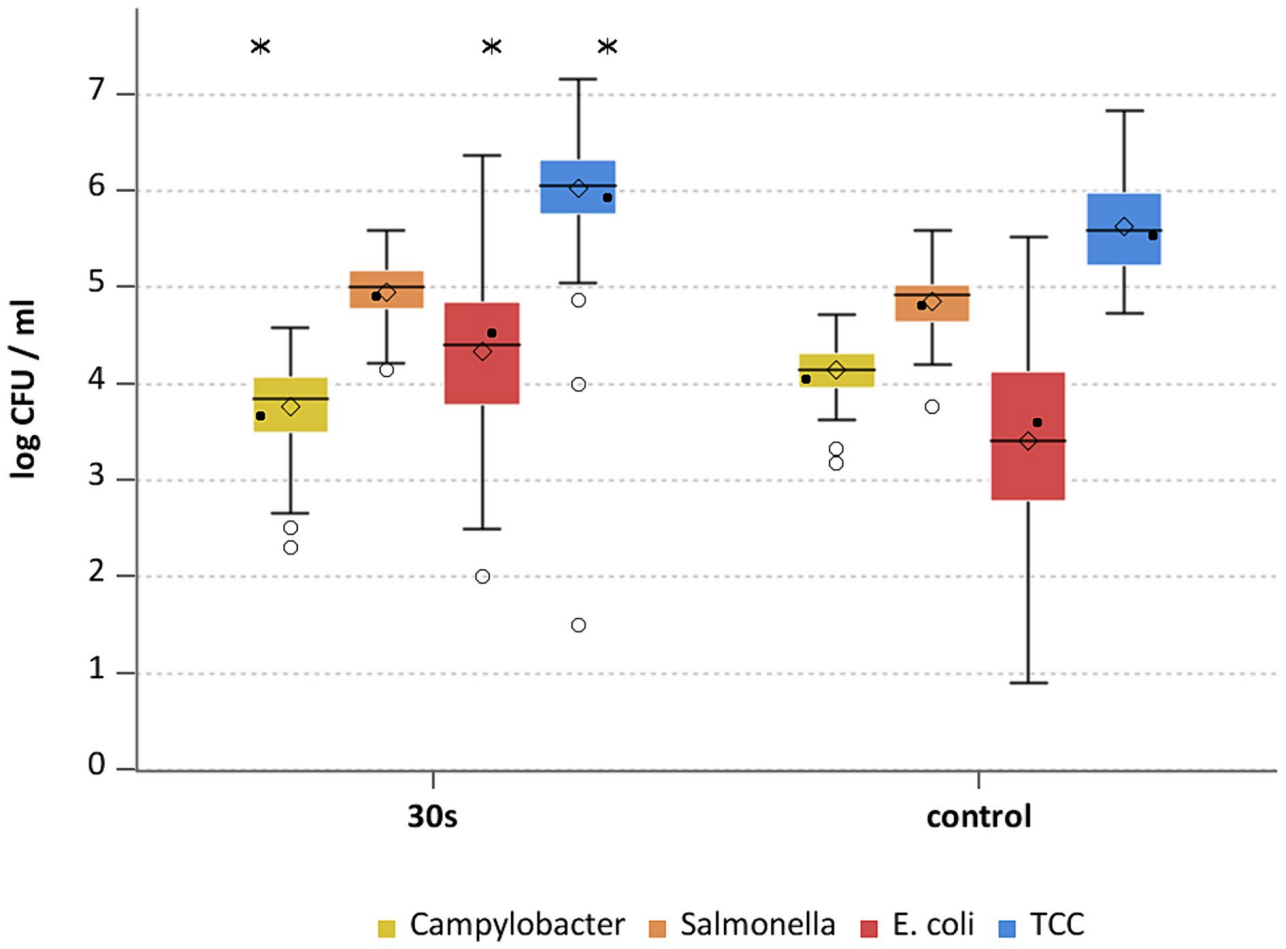
*Campylobacter* spp., *Salmonella* spp., *E. coli* and TCC levels of broiler carcasses after hot water immersion at 20°C for 30 s and corresponding untreated control group. Within box plots show the median (^___^), the least square mean (•), mean (◊) and outliers (o) are marked separately; * indicate statistically significant difference between treated groups and control group (*p* < 0.05, Tukey), (*n* = 102).

**Figure 6:**
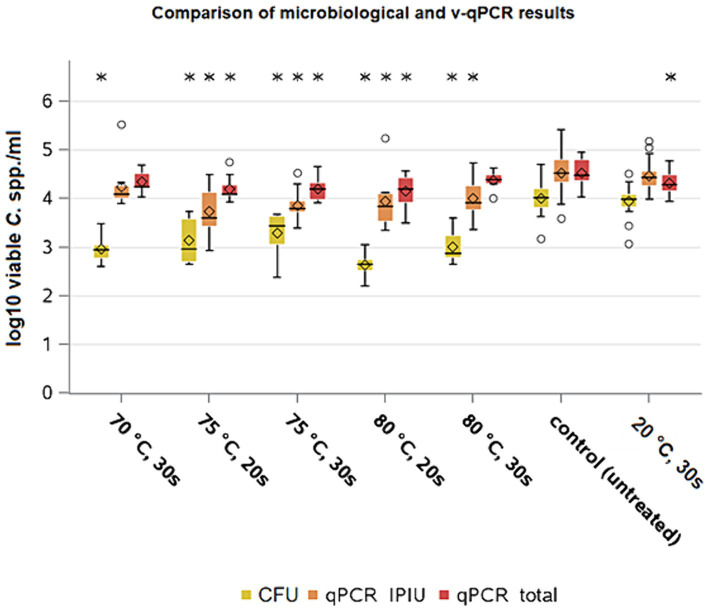
*Campylobacter* spp. counts of microbiological analysis and v-qPCR; yellow = microbiological results; orange = qPCR results – IPIU; red: qPCR results – total; Within boxplots show the median (^___^), mean (◊) and outliers (o) are marked separately, interquartile range and 95% confidence interval; * indicate statistically significant difference between treated groups and control group (*p* < 0.05, Tukey); (70 ° C: *n* = 10; 75°c 20s: *n* = 11; 75°C 30s: *n* = 10; 80°C 20s: *n* = 8; 80°C 30s: *n* = 8; 20°C 30s: *n* = 24; control: *n* = 39).

**Figure 7:**
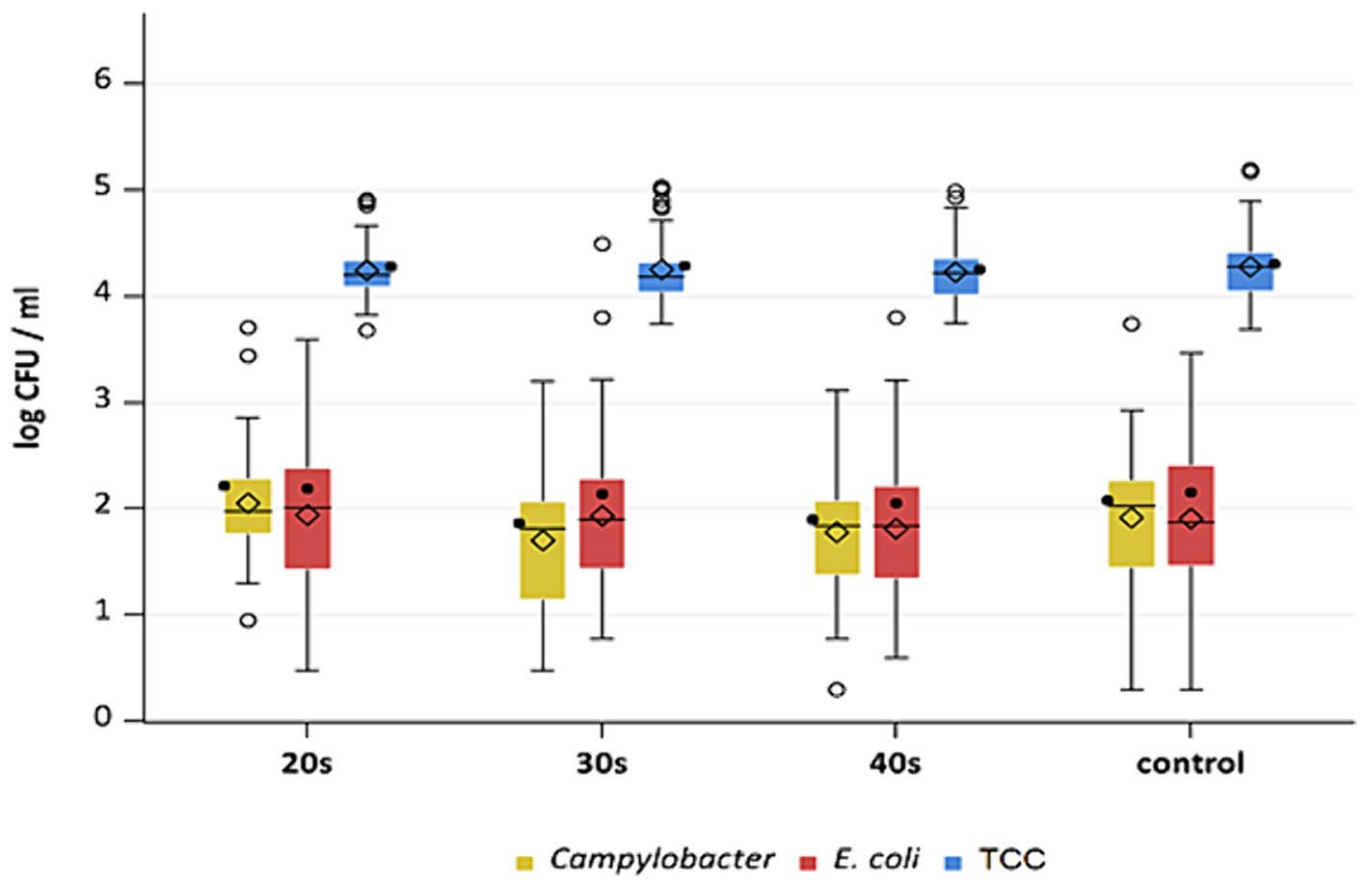
*Campylobacter* spp., *E. coli* and TCC levels of broiler carcasses at −80°C after 20, 30 and 40 s of cold air treatment and control group (untreated). Within boxplots show the median (^___^), the least square mean (•), mean (◊) and outliers (o) are marked separately, interquartile range and 95% confidence interval; * indicate statistically significant difference between treated groups and control group (*p* < 0.05, Tukey) (*n* = 51).

**Figure 8:**
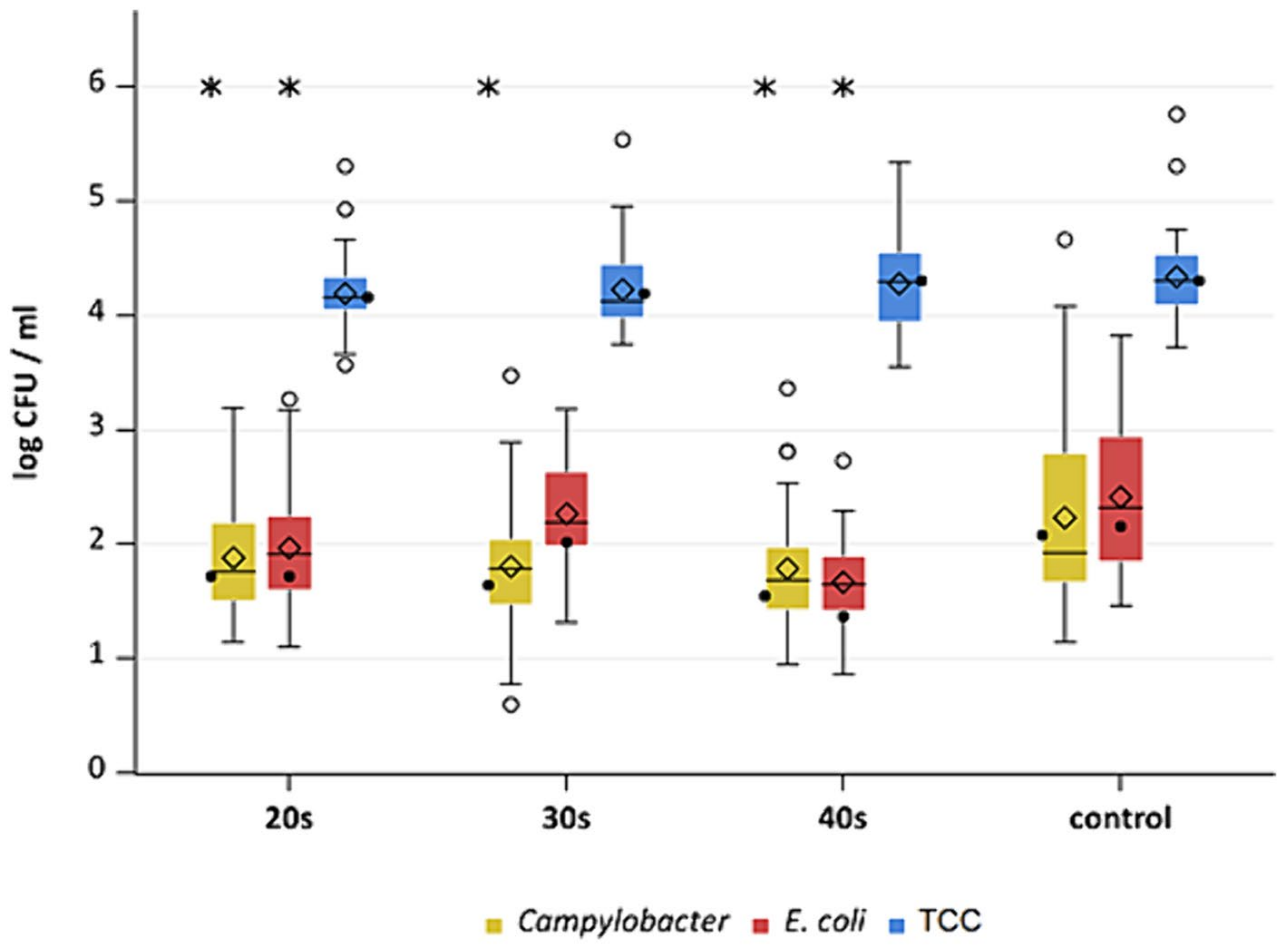
*Campylobacter* spp., *E. coli* and TCC levels of broiler carcasses at −90°C after 20, 30 and 40 s of cold air treatment and control group (untreated). Within boxplots show the median (^___^), the least square mean (•), mean (◊) and outliers (o) are marked separately, interquartile range and 95% confidence interval; * indicate statistically significant difference between treated groups and control group (*p* < 0.05, Tukey), (*n* = 51).

**Figure 9:**
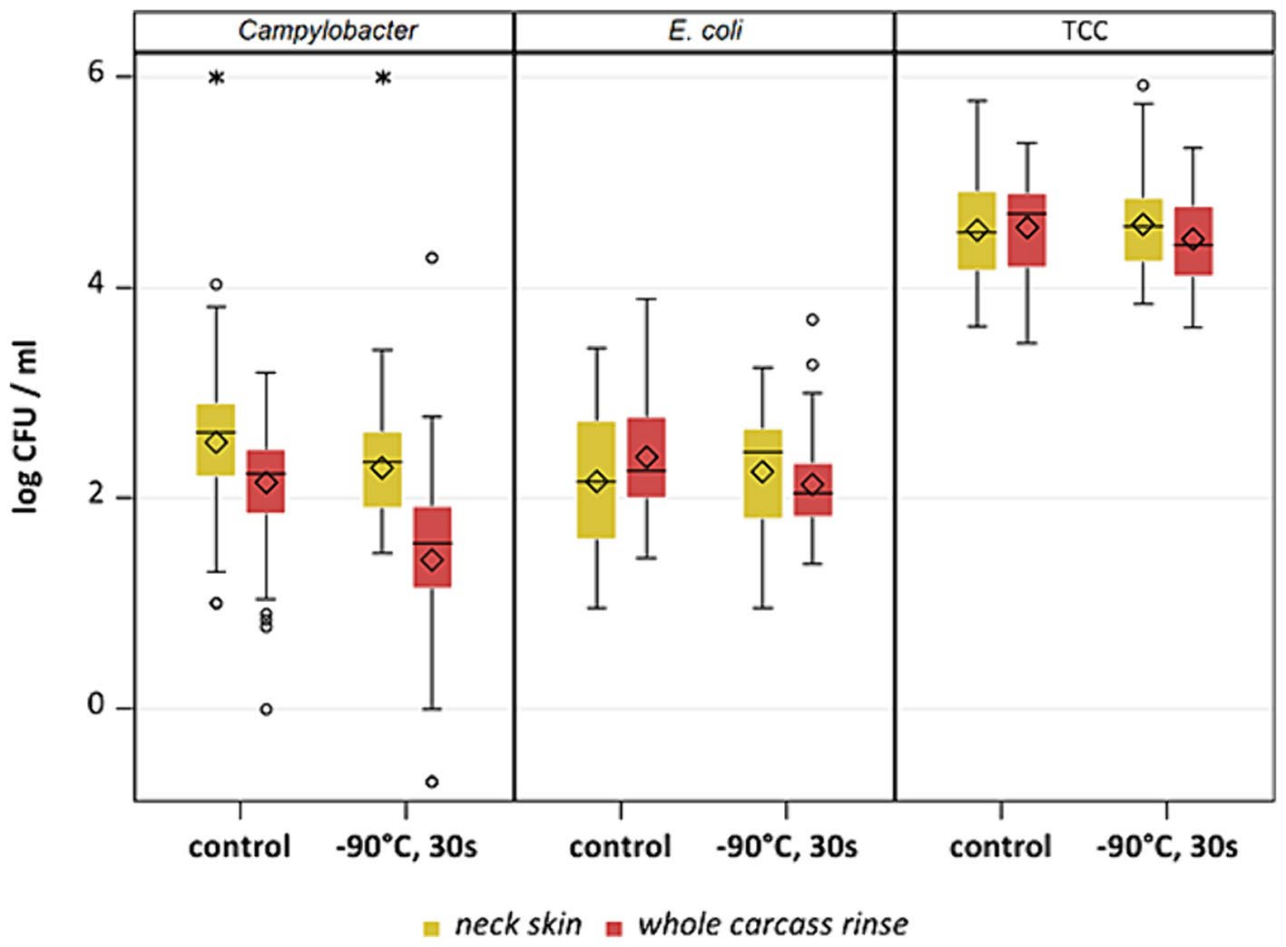
Comparison of neck skin and WCR after −90°C 30s cold-air treatment and untreated (control) for *Campylobacter*, *E. coli* and TCC; Within boxplots show the median (^___^), mean (◊) and outliers (o) are marked separately, interquartile range and 95% confidence interval; * indicate statistical difference between groups (*p* < 0.05, Tukey).

Since not all trial settings were conducted equally, e.g., the 70°C setting for the hot water treatment was not conducted for 20 s, temperature and exposure time, were combined into one variable. This enabled us to perform pairwise comparisons for all trial settings. The date of sampling was included in all models and was adjusted for using the least square means (LSM) method, since all temperature trials were conducted on different sampling days, whereas the control and room temperature samples were taken on all days. Results from the ANOVA and additional descriptive tables are displayed in Supplementary Tables S1–S6. Additionally, the LSM was included in the Figures 2–9, where each temperature setting is displayed with their respective control group. For groups where the LSM was statistically significantly different (*p* < 0.05) from the control group, a * is displayed above boxplot.

As for the comparison of microbiological methods, three different analyses were conducted. Firstly, ANOVA was performed for each method including date, temperature and time as fixed effects. As described above temperature and exposure time were combined into one variable for this analysis to compare the different trial settings for each method. Secondly, for pairwise comparison of methods, ANOVA with repeated measurements was performed. For better comparison of methods temperature, time and method were combined into one variable. The date of sampling was adjusted for as described above.

To assess the mean differences between neck skin and WCR samples and their controls, ANOVA was performed including the sample type (WCR or neck skin) and group (test or control) as fixed effects. To identify possible variation between the dates of sampling this was included as a random effect.

The *α*-level for all analyses was set to 5%. All statistical analyses were made with SAS®, version 9.4 (SAS Institute Inc., Cary, USA).

## 3 Results and discussion

### 3.1 Heat treatment

It was aimed to investigate the effectiveness of hot water treatment in reducing bacterial contamination with *Campylobacter* and *Salmonella* on broiler carcasses. The inoculation suspension with cecal contents displayed a mean concentration of 8.0 ± 0.3 log_10_ CFU/ml for *C. jejuni* and 8.4 ± 0.2 log_10_ CFU/ml for *Salmonella*. Bacterial mean counts for all untreated control groups were 4.1 ± 0.2 log_10_ CFU/ml WCR for *Campylobacter* spp. and 4.8 ± 0.3 log_10_ CFU/ml WCR for *Salmonella*. Expected concentrations which were calculated based on the measurement of the inoculation suspension were 4.4 ± 0.3 log_10_ CFU/ml for *Campylobacter* and 4.8 ± 0.2 log_10_ CFU/ml for *Salmonella*.

The general outcome of each treatment was a reduction of *Campylobacter* and *Salmonella,* where each temperature and exposure time had an effect (*p* < 0.05; Supplementary Table S3). A treatment at 70°C for 30 s for *Campylobacter* and *Salmonella* already showed mean reductions of 1.1 ± 0.5 and 0.7 ± 0.4 log_10_ CFU/ml (both *p* < 0.05), respectively (Figure 2). The trends for those bacteria were similar for the groups treated at 75°C and 80°C (Figures 3, 4), with a mean reduction of 1.0 ± 0.4 at 75°C for both exposure times (*p* < 0.05) and 1.4 ± 0.4 and 1.2 ± 0.7 log_10_ CFU/ml at 80°C (*p* < 0.05) for *Campylobacter* after 20 and 30 s, respectively. For *Salmonella* the treatments achieved a reduction of 0.9 ± 0.4 at 75°C for both exposure times and 1.1 ± 0.4 log_10_ CFU/ml for 80°C 20 s (*p* < 0.05) and 1.0 ± 0.7 log_10_ CFU/ml for 80°C 30 s (*p* < 0.05).

Whether the reducing effect was a result of the heat treatment alone or a combination of heat application and washing off by immersion into water was evaluated by an additional group of carcasses treated with water of approximately 20°C for 30 s. Comparing the untreated control group to the group rinsed at room temperature, the rinsing *per se* resulted in different effects for the inoculated pathogens and hygiene indicators compared to the control group (Supplementary Table S3). The difference on the inoculated bacteria were small for *Campylobacter*, with a mean reduction of 0.4 ± 0.5 log_10_ CFU/ml (*p* < 0.05), *Salmonella* remained at a similar level (Figure 5).

Heat treatment is already a part the slaughtering process of broilers in form of scalding, either with water immersion or steam/air, mainly, to loosen the feather follicles to improve the defeathering and additionally to reduce the bacterial contamination of the carcasses (Lu et al., 2019). Using hot water immersion for broiler carcasses directly after evisceration with temperatures of 70 to 80°C and exposure times of 20 and 30 s reduced the *Campylobacter* and *Salmonella* counts of around 1 log_10_ CFU/ml on the carcasses in this study. The highest reductions were found for 80°C for both inoculated bacteria. Rinsing by immersion in water alone was not sufficient for a reduction. Corry et al. (2007) achieved the highest mean reduction of 1.66 log_10_ CFU/cm^2^ on broiler carcasses artificially contaminated with *Campylobacter* with treatment at 75°C for 30 s, treatments at 70°C for 40 s and at 80°C for 20 s were tested as well. Similar findings for *Campylobacter* were described by Purnell et al. (2004), who analyzed neck skin samples and whole carcass rinses of heat treated (between 65 and 80°C for 10 to 70 s) and then fully processed broiler carcasses after different storage times (0 to 10 days post-kill). Reductions around 1 log_10_ CFU/ml or CFU/g could be achieved with treatments at 75 and 80°C, respectively (Purnell et al., 2004). Whyte et al. (2003) investigated hot water immersion at 75, 80 and 85°C for 10 and 20 s on retailed broiler thighs which were either inoculated or naturally contaminated with *Campylobacter* spp. and found significant reductions of up to 1 log_10_ CFU/ml after treatment at all temperatures for the artificially and naturally contaminated samples. Kure et al. (2020) treated eviscerated broiler carcasses with steam at 95 or 120°C for 3 or 5 s, achieving a reduction of *C. jejuni* from 0.84–4.32 log_10_ CFU/ml and 1.36–3.05 log_10_ CFU/ml for *S.* Enteritidis on breast, legs and wings. Li et al. (2002) tested hot water spray at 55 and 60°C for 12 s. This treatment significantly reduced inoculated *Campylobacter* concentrations by more than 0.78 log_10_ CFU/carcass. A spray treatment at 20°C for 120 s, had no effect which is consistent with the results of the current study. Although the samples in this study were immersed into water and not sprayed. The reducing effect in this study can therefore be explained by the effect of heat. In the early 2000s, some hot water immersion studies were conducted, that differed from the recent study in terms of sample numbers, sample types and experimental design. Additionally, the two main broiler associated pathogens *Campylobacter* and *Salmonella* were analyzed in this study, as well as natively occuring hygiene indicators, i.e., *E. coli* and TCC. Moreover, the most recent findings indicate a consistent pattern in the impact of hot water, which is a notable advancement from previous research. The experimental scenario closely resembles industrial conditions, enhancing the reliability of the results. Reducing *Campylobacter* contamination on broiler carcasses as in the recent study can help to reduce the risk for the consumer (EFSA, 2011).

Heat treatment in combination with other decontamination measures can enhance the reduction of *Campylobacter* counts; for instance, steam (90–94°C) in combination with ultrasound (30–40 kHz, SonoSteam™; Musavian et al., 2014) or in combination with ultrasound and rapid cooling (James et al., 2007), resulted in reductions of *Campylobacter* on broiler carcasses and skin between 1.4 log_10_ CFU/carcass and 2.9 log_10_ CFU/ /cm^2^, respectively. Northcutt et al. (2005) used comparatively lower temperatures of 43.3 and 54.4°C and an exposure time of 5 s for spray washing in an inside-outside bird washer, which did not result in a significant effect on the concentrations of TCC, *E. coli* and *Campylobacter*. This underlines the observation that additional hot water treatment would need to exceed the standard scalding temperatures to achieve a reduction of pathogenic bacteria on the carcasses in short-time treatments.

#### 3.1.1 Comparing microbiological and molecular biological results - *Campylobacter*

The values of the molecular biological analysis of the control group counts showed a mean value of 4.6 ± 0.2 log_10_ CFU/ml (*n* = 39) for *Campylobacter*, which matched the calculated concentration after inoculation as shown above. The averages of the IPIUs for the heat-treated groups ranged between 3.7 and 3.9 log_10_ IPIU/ml. The effect of the heat treatment was significant for both exposure times with reductions of 0.5 log_10_ IPIU/ml at 70°C, 0.7 and 0.6 log_10_ IPIU/ml at 75°C and 0.7 log_10_ IPIU/ml at 80°C for both exposure times (*p* < 0.05) compared to the control group. The mean concentration of total DNA of *Campylobacter* in the group treated by immersion in water at 20°C was 4.3 ± 0.2 log_10_ CFU/ml in the qPCR (*n* = 24) and remained at a similar level as the control group (Figure 6). The quantification data from v-qPCR was based on the measured ct-values of the samples in relation to ct-values from a standard curve (Supplementary Figure S1). The mean (*n* = 12) slope for duplex v-qPCR 1 was −3.37 ± 0.18, i.e., v-qPCR efficiency of 98.0% and for duplex v-qPCR 2 slope − 3.41 ± 0.15, i.e., v-qPCR efficiency of 96.5%.

When comparing the analysis methods, a higher value by 0.5 log_10_ per ml (*p* < 0.05) was found on average for the IPIU qPCR of the control group compared to the CFU results, which was also seen for the group treated at 20°C (*p* < 0.05). For the cultural analysis, the values of the groups treated at 75 and 80°C were 3.2 and 2.7 and 2.9 log_10_ CFU/ml for 20 and 30 s each, respectively. So mean differences from 0.6 to 1.3 log_10_ CFU/ml (*p* < 0.05) were found between the two detection methods. The bacterial reduction for the heat treatments shown via v-qPCR was smaller (*p* < 0.05) than the reduction seen via culturing.

The v-qPCR method allowed the detection of viable but non-culturable (VBNC) *Campylobacter* cells. Thus, providing a new perspective on the effect of decontamination treatments helps to close the gap between results from microbiological and molecular biological analysis. Deviations in *Campylobacter* concentrations were also found for the control group when analyzed by both methods. As a lower recovery of *Campylobacter* by culturing compared to v-qPCR was seen for samples that were not additionally stressed by heat, but only influenced by the environmental factors and the sampling method.

As a note, due to contamination in some sample extraction controls, some samples were excluded from the evaluation after qPCR analysis and this resulted in different sample numbers for each group. Nevertheless, the results have been statistically assessed by fitting the models to the sample numbers.

The v-qPCR method by Stingl et al. (2021) applied in this study with the ability to detect non growing but still viable cells through live-dead differentiation, has only recently been published. There are only a few studies available to date comparing the data generated by both methods under various applications. For instance, it was reported that discrepancies of 1 log_10_ CFU/ml in *Campylobacter* counts between cultural and molecular biological methods were measured, when storing lamb or chicken meat under different packaging conditions (Beterams et al., 2023; Lazou et al., 2021; Wulsten et al., 2022). The difference in the results between the two analytical methods shows the limitations of culturing. Factors such as selective growth conditions, may underestimate bacterial counts while it can potentially lead to overestimating the effectiveness of an intervention. Non-differentiating qPCRs on the other hand may also detect dead cells what can result in an overestimating of potentially intact bacterial cells. Likewise, in our analysis, the total amount of *Campylobacter* spp. DNA in samples with various treatments was not always significantly different (Figure 6). The v-qPCR method can help to better evaluate the reducing effect of decontamination methods on bacteria. The reductions observed in both analytical methods highlighted heat as an additional decontamination treatment during broiler meat processing, although, the effectiveness was lower when qPCR results were considered.

#### 3.1.2 Hygiene indicators

Heat treatment showed different effects on *E. coli* and TCC. For *E. coli* the means of the treated groups were 3.6 ± 0.7 log_10_ CFU/ml (70°C), 3.2 ± 0.9 log_10_ CFU/ml and 3.3 ± 0.9 for 20 and 30 s (75°C) and 3.2 ± 0.9 log_10_ CFU/ml and 3 ± 0.7 log_10_ CFU/ml for 20 and 30 s (80°C). No statistically significant reductions were seen for hot water treatments for *E. coli* (Supplementary Table S3). Compared to the trial specific control group a reduction of TCC was observed for all settings (*p* < 0.05; Supplementary Table S3) with a maximum of 0.9 log_10_ CFU/ml at 75°C for both exposure times (Figures 2–4). A few of assumptions can be made regarding the reasons why a reduction in *E. coli* counts was not observed: (a) presence of *E. coli* in deeper skin layers and the associated adherence or protection from heat; (b) a different recovery of the bacteria between inoculated versus naturally occurring bacteria due to the water bath itself; (c) different physiological state and therefore heat tolerance of the native bacteria compared to the inoculated bacteria. Hauge et al. (2023) examined broiler carcasses for *E. coli* and TCC treated with hot water at 70, 80, and 90°C for 3 s and 80°C for 6 s. Only the 80°C and 6 s treatment resulted in a reduction in *E. coli* counts with a reduction of 1.1 log_10_ CFU/ml. TCC was reduced by 1–1.1 log_10_ CFU/ml at 80°C for both exposure times as well as at 90°C for 3 s. The results for *E. coli* differed in the recent study, whereas results for TCC were similar, even though the reductions did not increase with higher exposure times and treatment temperatures in this study.

Hygiene indicators were found to be increased after the immersion at room temperature compared to the untreated control group (*p* < 0.05). Values for *E. coli* and TCC were higher by 0.9 ± 1.2 and 0.4 ± 0.7 log_10_ CFU/ml (Figure 5). By wetting the surfaces before sampling, e.g., by immersion in a water bath, the poultry skin absorbs water and surface layers are opened (Selgas et al., 1993), so that bacteria can be washed off the skin more easily during the sample processing. For dry surfaces, i. e. the control group in this study, Hutchison et al. (2022) assumed, that bacteria are more strongly attached and are therefore harder to remove from the chicken skin with WCR. These assumptions could explain the observation in this study.

### 3.2 Cold air treatment

This study aimed to investigate the effectiveness of cold air treatment in reducing natural *Campylobacter* contamination on broiler carcasses before chilling. Several temperatures and exposure times were tested, in particular −80°C and − 90°C for 20, 30, and 40 s (Figures 7, 8). Overall, a reduction of *Campylobacter* (*p* < 0.05; Supplementary Table S6) was seen only for −90°C, the mean reduction was 0.4 ± 0.9 log_10_ CFU/ml, 0.4 ± 1 log_10_ CFU/ml and 0.5 ± 0.8 log_10_ CFU/ml at a treatment time of 20, 30 and 40 s (*p* < 0.05; Supplementary Table S6), respectively (Figure 8). For the −90°C and 30 s treatment, additional trials were conducted as part of the comparison analysis for WCR and neck skin. In those trials, a mean reduction of *Campylobacter* of 0.7 log_10_ CFU/ml (p < 0.05) was observed for the WCR, from an initial mean concentration of the untreated groups of 2.1 log_10_ CFU/ml (Figure 9).

Based on the observation of the data from the individual trials it seemed that the initial carcass contamination with *Campylobacter* corresponded with the reducing effect. There was a hint for a larger reduction when contamination was higher (Tables 1, 2), because the individual mean concentrations of *Campylobacter* on untreated control carcasses were higher in the batches for trials at −90°C with 2.2 ± 0.4 log_10_ CFU/ml, whereas batches treated at −80°C had contamination levels of 1.9 ± 0.7 log_10_ CFU/ml. The cooling of meat after slaughtering is important to prevent the proliferation of bacteria and to extend the shelf life of the meat, there is also a potential for bacteria reduction (Kennedy and Miller, 2004). In the EU, different chilling methods are used at broiler slaughterhouses, such as air, immersion or a combination cooling (Varzakas and Feddern, 2016). Moreover, freezing (−18 to −22°C) was also described as a method to reduce the bacterial load, especially of temperature-sensitive bacteria, such as *Campylobacter* spp. (Harrison et al., 2013; Sampers et al., 2010). But freezing is not an option in the fresh meat market because of the European law for fresh poultry meat (regulation (EC) 543/2008, Anonymous, 2008). A cold treatment beyond the normal cooling process can additionally be used to improve the reduction of the microbial load. Short-time treatment with very cold air, as investigated in the current study, results in superficial freezing of carcasses and showed a bacteria-reducing potential for *Campylobacter* of up to 0.5 log_10_ CFU/carcass in pilot studies by Boysen and Rosenquist (2009). Haughton et al. (2012) used so called crust freezing with different combinations of temperature and time alone and in combination with UV light irradiation on chicken lower legs and in both cases a significant reduction of the *Campylobacter* load of 0.5 to 1.0 log_10_ CFU/g skin was demonstrated. In another study, cooling broiler skin of fresh drumsticks in liquid nitrogen to a minimum surface temperature of −17°C for up to 10 s and spraying broiler carcasses with liquid nitrogen for 40 s reduced *Campylobacter* concentrations by 0.9 and 1.5 log_10_ CFU/g skin (Burfoot et al., 2016). In the United Kingdom, the indirect cryogenic surface chilling method is already used in practice at a number of slaughterhouses (Herrmann et al., 2017).

**Table 1:** *Campylobacter*, *E. coli* and TCC on broiler carcasses pre-chilling treated with −80°C for different exposure times (20, 30, 40s), mean values ± SD.

Temperature (°C)	Trial	Time (s)	*Campylobacter*		*E. coli*		TCC	
			Mean	± SD	Mean	± SD	Mean	± SD
−80	1	20	2.0	0.2	2.3	0.4	4.2	0.1
		30	2.0	0.6	2.3	0.5	4.2	0.3
		40	2.1	0.5	2.3	0.5	4.3	0.2
		Control	2.5	0.4	2.0	0.6	4.2	0.4
−80	2	20	1.8	0.4	1.2	0.5	4.2	0.2
		30	1.1	0.5	1.4	1.0	4.4	0.4
		40	1.4	0.6	1.1	0.5	4.2	0.3
		Control	1.2	0.4	1.4	0.6	4.4	0.3
−80	3	20	2.4	0.6	2.3	0.7	4.4	0.3
		30	2.0	0.2	1.9	0.4	4.2	0.3
		40	1.9	0.4	2.0	0.6	4.2	0.3
		Control	2.1	0.4	2.0	0.5	4.3	0.3

**Table 2:** *Campylobacter*, *E. coli* and TCC on broiler carcasses pre-chilling treated with −90°C for different exposure times (20, 30, 40s), mean values ± SD.

Temperature (°C)	Trial	Time (s)	*Campylobacter*		*E. coli*		TCC	
			Mean	± SD	Mean	± SD	Mean	± SD
−90	1	20	2.2	0.5	1.9	0.6	4.2	0.3
		30	2.1	0.6	2.7	0.3	4.1	0.3
		40	2.0	0.6	1.5	0.4	4.2	0.5
		Control	2.8	1.0	3.0	0.5	4.3	0.5
−90	2	20	1.7	0.6	2.0	0.6	4.2	0.5
		30	1.7	0.6	2.0	0.4	4.5	0.5
		40	N/A	N/A	N/A	N/A	N/A	N/A
		Control	2.1	0.6	2.3	0.6	4.5	0.4
−90	3	20	18	0.3	2.0	0.4	4.2	0.2
		30	1.8	0.5	2.1	0.4	4.1	0.3
		40	1.7	0.4	1.8	0.4	4.3	0.2
		Control	2.1	0.6	2.0	0.5	4.3	0.2

The cold air treatment is a fast treatment, which did not affect the sensory appearance of the carcasses. The initial contamination of carcasses was reduced during the routine slaughtering process, to mean levels of *Campylobacter* of around 2 log_10_ CFU/ml of carcass rinse after chilling (Beterams et al., 2024). The additional reduction of the SafeChill™ cold air treatment appeared to be linked to the level of contamination, detection method and the limit of detection. If the contamination was higher, the decontamination effect seemed to be larger.

#### 3.2.1 Hygiene indicators

For the cold-treated groups, *E. coli* values ranged between 1.7 ± 0.4 and 2.3 ± 0.5 log_10_ CFU/ml. A reduction (*p* < 0.05) of 0.4 ± 0.9 was observed for 20 s at −90°C and 0.8 ± 1 log_10_ CFU/ml for 40 s at −90°C. Mean values for TCC ranged from 4.2 to 4.3 ± 0.3 log_10_ CFU/ml for the treated and untreated groups and were not affected by the cold treatment (Figures 7, 8). Those observations were further confirmed by the additional trials conducted at −90°C for 30 s (Figure 9). As for control groups, the mean levels were 1.9 ± 0.7 for −80°C and 2.4 ± 0.7 log_10_ CFU/ml for −90°C for *E. coli*. The decontaminating effect on *E. coli* seemed to be larger when the colony counts were higher. In contrast, Chaves et al. (2011) described a process, where crust freezing at −85°C was used for 20 and 60 min and did not result in a reduction of *E. coli* counts.

#### 3.2.2 Comparing analysis of neck skin samples and WCR

Different sampling methods used for broiler carcasses can influence the enumeration results. In a previous study, differences in the level of *Campylobacter* between neck skin and WCR were identified after chilling (Beterams et al., 2024). To be able to evaluate this under additional treatment, the two sample types were compared within the cold air study. Overall, the bacterial reduction of the cold air treatment differed between the sample types. We compared the results for neck skin and WCR samples with each other after cold-air treatment at −90°C. Counts of *Campylobacter* were higher for the neck skin samples (p < 0.05) with means between 2.3 and 2.5 log_10_ CFU/g than for the WCR samples with mean values between 1.4 and 2.1 log_10_ CFU/ml. The values for WCR were lower even before the treatment after pre-chilling compared to neck skin samples. Based on the neck skin samples, the cold treatment had no effect on *Campylobacter*, while by analysis of WCR a mean reduction of 0.7 log_10_ CFU/ml was observed (Figure 9). For *E. coli*, the samples for all groups showed high variability in the results, an effect was only recognizable for treatment using WCR (p < 0.05) with a reduction of 0.3 log_10_ CFU/ml compared to the control group.

Nagel Gravning et al. (2021) compared four different sampling methods with each other, converted the results into log CFU/cm^2^ (WCR, neck skin, breast skin, swabbing), and identified WCR as the most effective method to recover *E. coli* with the lowest variation in values and the highest recovery rate. For TCC, the levels for neck skin samples and WCR were comparable. In the current study, these assumptions could be underlined completely, only *E. coli* showed higher values in mean for the WCR for the control groups, TCC remained on the same level for both sampling types. Also, for *Campylobacter*, neck skin samples showed higher concentrations than WCR (Figure 9). The drying of the carcass surface during chilling can influence the recovery of bacteria by rinsing and lead to a possible underestimation of colony counts on the carcass (Hutchison et al., 2022). This could explain the lower recovery of WCR compared to neck skin samples, which was also described by Beterams et al. (2024). Moreover, the distribution of bacteria on different areas of the carcass´ skin can have an influence on the enumeration results of the different sample types. Smith (2010) also found significantly higher recovery rates for naturally occurring *E. coli* in rinsed samples than in ground, which was also represented in the current study.

### 3.3 Sensory analysis

**Figure 10:**
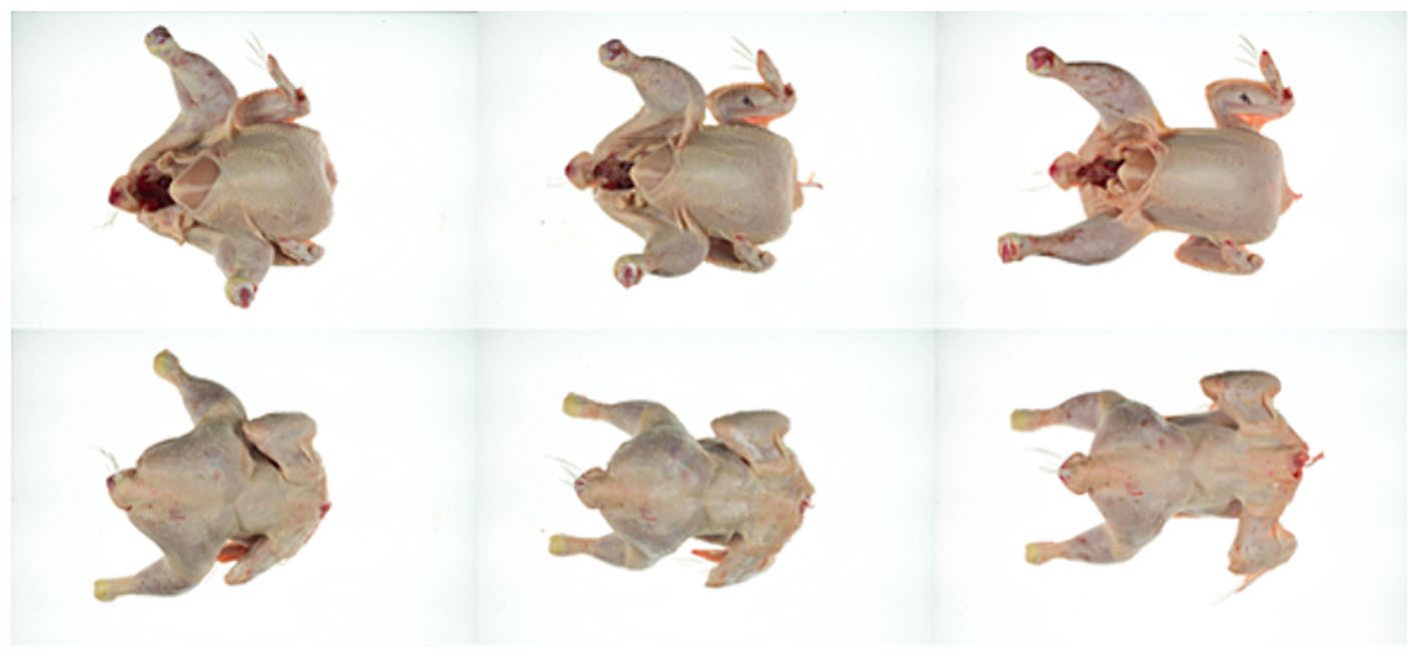
Example of a carcass treated at 75°C for 20 s; before treatment (left), after treatment (center) and after air chilling (right), viewed from the front (top) and back (bottom).

**Figure 11:**
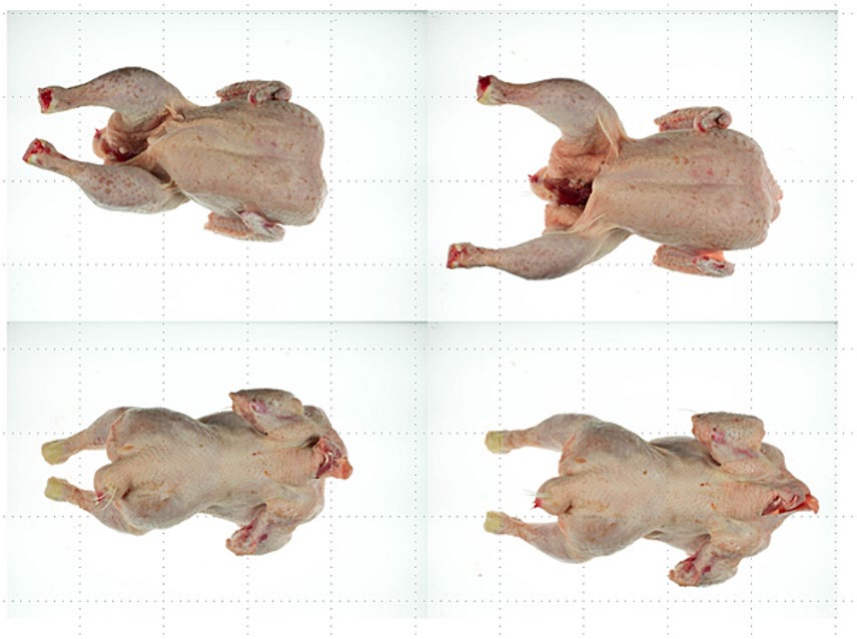
Example of a carcass without treatment (left) and after air chilling (right), viewed from the front (top) and back (bottom).

When meat or skin are treated with hot water, a change in appearance is possible. The visual appearance and the texture of the skin was therefore evaluated before and after treatment at 70°C for 30 s, at 75 and 80°C for 20 and 30 s each and after additional air chilling for 2 h (*n* = 3). The skin of untreated carcasses had an elastic, soft but strong consistency that needed some force to be ripped apart. After treatment, the skin texture became more inflexible, tough and needed less force to be ripped apart. This effect became increasingly apparent with higher temperature and longer exposure time. The visual appearance was also slightly different for the untreated and treated groups with a tendency for the skin to appear cooked (slightly lighter) in particular for carcasses treated at 80°C (Figures 10, 11). The temperature beneath the skin was at a maximum of 60°C for the hottest and longest treatment (80°C, 30 s), but after heat treatment the meat beneath the skin did not show deviations in appearance compared to the untreated samples (Table 3). Using colorimetric measurement of the carcass skin, the L*-characteristic did not appear to change with treatment alone. Values of untreated and treated carcasses were between L* 70.0 ± 1.0 and L* 71.3 ± 1.7 (Table 3). Whereas after chilling, L*-values showed a tendency for increased values on both untreated or treated carcasses. Chilled carcasses showed an increase in yellowness (visual appearance supported by data) of the skin and a tendency for a larger delta in b* (+)-values for the heat treatment (70°C, 30 s: b* (+) 7.4 ± 0.8; 80°C, 30 s: b* (+) 12.1 ± 1.5).

**Table 3:** L, a (+) and b (+) values mean and standard deviation for broilers of the different treatment groups.

Temperature (°C)	time (s)	Condition		L*	a* (+)	b* (+)
70	30	a	Mean	69.3	7.0	5.4
			SD	0.8	1.5	1
		b	Mean	70.0	6.6	6.0
			SD	1	1.5	0.8
		c	Mean	71.2	8.0	7.4
			SD	1.3	2.0	0.8
		d	Mean	62.7	12.5	5.0
			SD	2.8	1.7	2
75	20	a	Mean	71.5	8.3	8.3
			SD	2.1	0.8	0.7
		b	Mean	70.7	8.0	9.0
			SD	1.6	1.2	0.8
		c	Mean	70.9	9.9	11.3
			SD	1.9	1.5	1.2
		d	Mean	64.5	11.7	7.3
			SD	1.7	0.8	0.9
	30	a	Mean	71.2	8.2	8.7
			SD	1.6	1.4	1.1
		b	Mean	71.2	8.2	10.1
			SD	2.9	1.6	1.8
		c	Mean	70.7	8.9	10.0
			SD	3.4	2.3	1.2
		d	Mean	64.5	11.5	6.9
			SD	1.9	1.4	1.7
80	20	a	Mean	74.1	8.8	8.0
			SD	1.7	1.2	2.4
		b	Mean	71.1	8.2	9.4
			SD	1.5	0.8	1.7
		c	Mean	71.5	9.3	12.1
			SD	1.3	1.3	2
		d	Mean	66.6	10.4	7.3
			SD	1.1	0.4	1.1
	30	a	Mean	69.8	9.4	8.1
			SD	1.9	1.5	2.9
		b	Mean	70.8	8.7	9.9
			SD	2	1.0	1.9
		c	Mean	70.2	9.7	12.1
			SD	1.9	0.8	1.4
		d	Mean	66.0	11.1	7.3
			SD	1.2	1.1	1.6
Control		a	Mean	72.2	8.3	8.7
			SD	2.2	1.8	1.4
		c	Mean	74.3	8.8	10.1
			SD	2.9	2.2	1.6
		d	Mean	64.9	11.5	6.9
			SD	1.5	1.1	0.6

L* = lightness and darkness (+ lighter, − darker); a* (+) = red; b* (+) = yellow; conditions: a = untreated; b = after hot water treatment; c = after 2 h air chilling; d = after 2 h of air chilling, without breast skin (*n* = 5).

The use of thermal processes, especially when operating with temperatures outside the nowadays commonly used range for scalding and chilling, could result in changes in carcass appearance that could affect the acceptability of the final product. In Germany, around 83% of the produced amount of broiler meat is sold fresh, relevant parts of the broiler are legs and skinless chicken breast (Federal Ministry of Food and Agriculture, 2022). If decontamination treatments such as heat led to deviations in the appearance of the skin, they can more likely be applied to the parts that are marketed without skin.

Li et al. (2002) analyzed hot water spray at different temperatures on carcasses in an inside-outside washer and did not see changes in skin color even with the highest temperature set to 60°C. Similar observations were reported by Northcutt et al. (2005), who found no change in color of the skin at a maximum of 60°C and a treatment for 5 s. Thomson et al. (1974) on the other hand studied spray washing of broiler carcasses with hot water and reported “inferior appearance” of carcasses washed at 71.1°C for 12 s, although the magnitude of color difference was small. Purnell et al. (2004) found that treatment of broiler carcasses at 70°C for 40 s and storage afterwards under typical conditions for 8 days were effective to reduce *Campylobacter* concentrations without affecting chicken skin. In addition, Hauge et al. (2023) studied a 3 and 6 s treatment at 70 to 90°C and also found no visual change in skin texture and color, which could mainly be explained by the short treatment times. Colorimetric measurements in this current study showed a tendency to a slight increase of L*-values after chilling for both treated and untreated groups. Shung et al. (2022) compared two scalding regimes in Taiwan and evaluated “soft scalding” at 57°C for 120 s and “hard scalding” at 60°C for 60 s. Hard scalding resulted in an increase of yellowness (b* (+)) and lightness (L*). An increase in b* (+)-values was also observed within the current study.

For the cold air treatment, the colorimetric values for L*, a* (+) and b* (+) did not differ between untreated and treated carcasses after chilling (data not shown). The temperature under the skin was between −1 and 1°C in the breast area and between −0.5 and 1°C for the upper leg measured directly after the treatment. The lowest temperature in deeper muscle layers was 1°C. When treating carcasses using ultra cold air, it should be noted that freezing of the meat might occur. According to regulation (EU) No. 1308/2013 (Anonymous, 2013), the temperature of fresh chicken meat must not fall below −2°C. This must be considered in the potential application of those methods.

## 4 Conclusion

Hot water and cold air treatments of different temperatures and exposure times resulted in the reduction of bacterial contamination of broiler carcasses, i.e., *Campylobacter* and *Salmonella*. The hot water application at 70°C resulted in the reduction of target bacteria with only mild effects on the sensory appearance on the carcasses. So, physical treatments can lead to an additional reduction of *Campylobacter* and *Salmonella* by the end of primary processing as determined within this study. This can help to reduce the risk of foodborne infection from broiler meat. Both treatments can easily be implemented into industrial settings. However, decontamination efficacy needs to be verified under practical conditions. Molecular biological analysis for *Campylobacter* showed a statistically significant smaller reducing effect compared to culturing methods. By using different methods for the counting of bacteria in the analysis of decontamination measures, it became obvious that the reducing effect on viable cells of *Campylobacter* can be overestimated by using cultural quantification only. This method is still new and has not yet been applied in studies on the decontamination of *Campylobacter*. A wider application of molecular biological analysis using v-qPCR will provide additional information on the efficacy of decontamination treatments in the future.

## Data Availability

The raw data supporting the conclusions of this article will be made available by the authors, without undue reservation.

## Supplementary material

SUPPLEMENTARY FIGURE S1 Representative standard curves and equations for quantification of *Campylobacter* by v-qPCR according to the protocol by Stingl et al. (2021). The DNA standards of *C. jejuni* NCTC 11168 and *C. sputorum* DSM 5363 were measured in duplicate at copy numbers ranging from 1 to 3.7 log_10_ *C.* spp. genome equivalents. Duplex 1 (blue), 16S rRNA target of C. jejuni and internal amplification control; Duplex 2 (orange), 16S rRNA target of ISPC (*C. sputorum*) and internal amplification control; Cj, *C. jejuni*; ISPC, internal sample process control. The standard equations are depicted in the respective colour with x, log_10_ C. spp. genome equivalent/PCR well; y, ct value; R2, correlation coefficient.
