# Supplementary figures and images for: Application of hot water and cold air to reduce bacterial contamination on broiler carcasses
Source: Front Microbiol. 2024 Sep 19;15:1429756. doi: 10.3389/fmicb.2024.1429756 (PMC11457684; doi:10.3389/fmicb.2024.1429756)

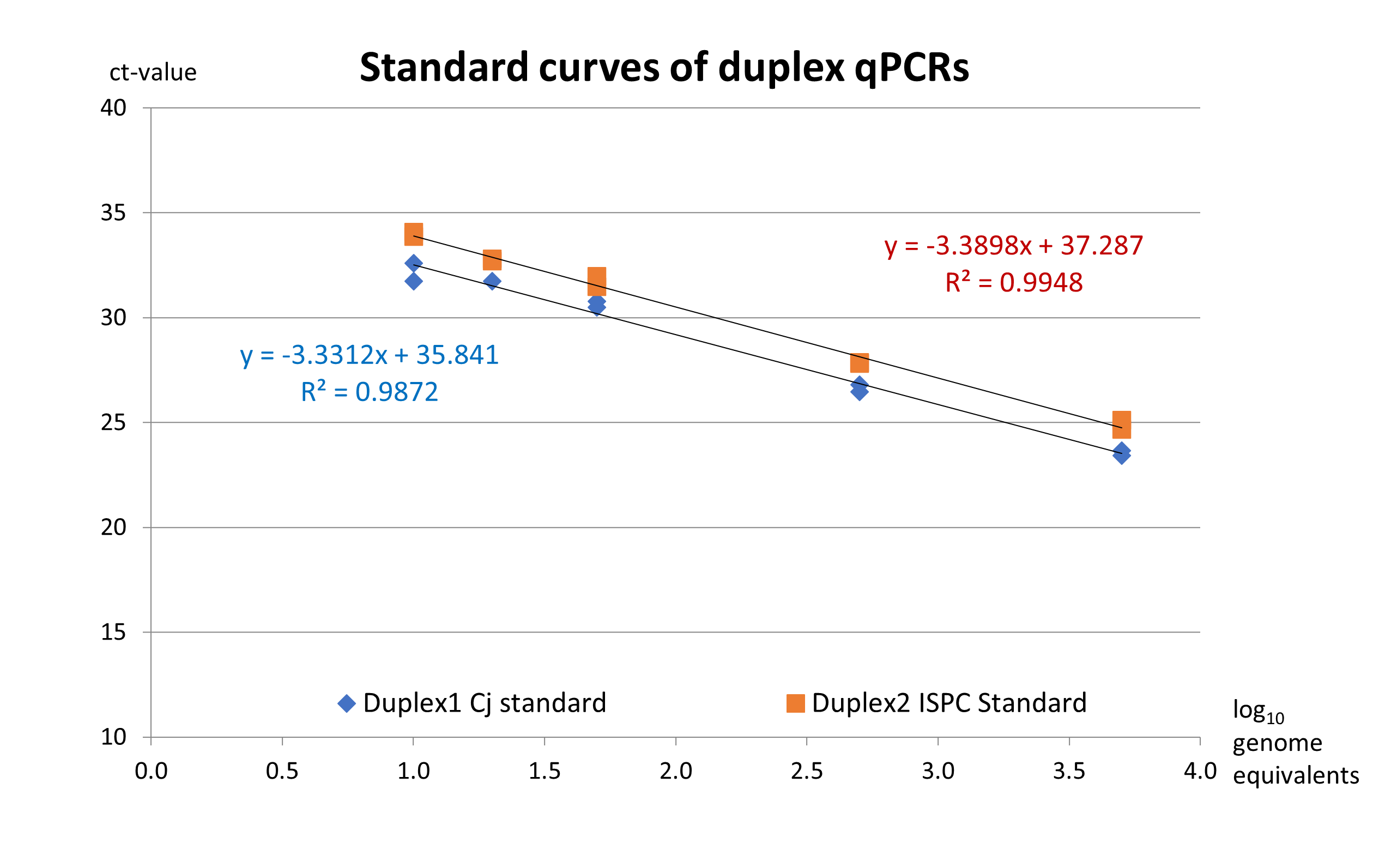

Supplement: SUPPLEMENTARY FIGURE S1 — Representative standard curves and equations for quantification of Campylobacter by v-qPCR according to the protocol by Stingl et al. (2021). The DNA standards of C. jejuni NCTC 11168 and C. sputorum DSM 5363 were measured in duplicate at copy numbers ranging from 1 to 3.7 log10 C. spp. genome equivalents. Duplex 1 (blue), 16S rRNA target of C. jejuni and internal amplification control; Duplex 2 (orange), 16S rRNA target of ISPC (C. sputorum) and internal amplification control; Cj, C. jejuni; ISPC, internal sample process control. The standard equations are depicted in the respective colour with x, log10 C. spp. genome equivalent/PCR well; y, ct value; R2, correlation coefficient. [file Image_1.TIF]
