# Supplementary material for: Application of hot water and cold air to reduce bacterial contamination on broiler carcasses
Source: Front Microbiol. 2024 Sep 19;15:1429756. doi: 10.3389/fmicb.2024.1429756 (PMC11457684; doi:10.3389/fmicb.2024.1429756)
Supplement: Supplementary file 2 [file Table_1.docx]

**Supplementary Data**

Suppl. Table 1: Descriptive statistics per trial setting for the hot water treatment

| Bacterium | Trial setting | N | Mean | Median | SD | CV | Min. | Max. | N Missing |
| --- | --- | --- | --- | --- | --- | --- | --- | --- | --- |
| *Campylobacter* | 70°C, 30s | 51 | 2.8 | 2.8 | 0.4 | 16.1 | 1.4 | 3.9 | 0 |
|  | control | 51 | 3.9 | 3.9 | 0.2 | 5.7 | 2.9 | 4.2 | 0 |
|  | 75°C, 20s | 51 | 3.2 | 3.2 | 0.4 | 13.7 | 2.6 | 4.1 | 0 |
|  | 75°C, 30s | 51 | 3.2 | 3.1 | 0.3 | 10.8 | 2.4 | 3.7 | 0 |
|  | control | 51 | 4.2 | 4.2 | 0.3 | 7.8 | 3.2 | 4.7 | 0 |
|  | 80°C, 20s | 51 | 2.7 | 2.6 | 0.4 | 13.6 | 2.0 | 3.6 | 0 |
|  | 80°C, 30s | 51 | 2.9 | 2.9 | 0.5 | 18.5 | 0.5 | 3.8 | 0 |
|  | control | 51 | 4.1 | 4.0 | 0.2 | 5.5 | 3.6 | 4.7 | 0 |
|  | 20°C, 30s | 102 | 3.8 | 3.8 | 0.5 | 12.9 | 2.3 | 4.6 | 0 |
|  | control | 102 | 4.1 | 4.1 | 0.3 | 6.9 | 3.2 | 4.7 | 0 |
| *E. coli* | 70°C, 30s | 51 | 3.6 | 3.6 | 0.7 | 18.2 | 2.1 | 5.4 | 0 |
|  | control | 51 | 4.0 | 4.0 | 0.7 | 18.0 | 2.6 | 5.6 | 0 |
|  | 75°C, 20s | 51 | 3.2 | 3.2 | 0.9 | 27.4 | 1.7 | 5.4 | 0 |
|  | 75°C, 30s | 51 | 3.3 | 3.1 | 0.8 | 25.3 | 1.4 | 5.0 | 0 |
|  | control | 51 | 3.5 | 3.4 | 1.0 | 27.8 | 2.0 | 5.5 | 0 |
|  | 80°C, 20s | 51 | 3.2 | 3.1 | 0.8 | 26.4 | 1.8 | 5.1 | 0 |
|  | 80°C, 30s | 51 | 3.0 | 2.9 | 0.7 | 24.3 | 1.6 | 5.4 | 0 |
|  | control | 51 | 3.4 | 3.4 | 0.9 | 27.4 | 0.9 | 5.3 | 0 |
|  | 20°C, 30s | 102 | 4.3 | 4.4 | 0.9 | 20.0 | 2.0 | 6.4 | 0 |
|  | control | 102 | 3.4 | 3.4 | 0.9 | 27.6 | 0.9 | 5.5 | 0 |
| *Salmonella* | 70°C, 30s | 51 | 4.0 | 4.0 | 0.4 | 9.5 | 2.9 | 4.8 | 0 |
|  | control | 51 | 4.7 | 4.8 | 0.2 | 5.2 | 4.2 | 5.1 | 0 |
|  | 75°C, 20s | 51 | 4.0 | 4.0 | 0.5 | 12.7 | 3.1 | 5.3 | 0 |
|  | 75°C, 30s | 51 | 4.0 | 4.1 | 0.4 | 9.7 | 2.6 | 4.7 | 0 |
|  | control | 51 | 4.9 | 4.9 | 0.3 | 6.6 | 3.8 | 5.6 | 0 |
|  | 80°C, 20s | 51 | 3.8 | 3.8 | 0.5 | 14.5 | 2.3 | 4.9 | 0 |
|  | 80°C, 30s | 51 | 3.8 | 4.0 | 0.7 | 17.0 | 0.5 | 4.8 | 0 |
|  | control | 51 | 4.8 | 4.9 | 0.4 | 7.4 | 4.2 | 5.5 | 0 |
|  | 20°C, 30s | 102 | 4.9 | 5.0 | 0.3 | 6.5 | 4.2 | 5.6 | 0 |
|  | control | 102 | 4.9 | 4.9 | 0.3 | 7.0 | 3.8 | 5.6 | 0 |
| TCC | 70°C, 30s | 51 | 4.8 | 4.7 | 0.4 | 7.8 | 4.2 | 5.8 | 0 |
|  | control | 51 | 5.4 | 5.2 | 0.4 | 7.5 | 4.7 | 6.5 | 0 |
|  | 75°C, 20s | 51 | 4.8 | 4.8 | 0.6 | 11.6 | 3.9 | 6.1 | 0 |
|  | 75°C, 30s | 51 | 4.8 | 4.8 | 0.5 | 11.0 | 4.0 | 6.6 | 0 |
|  | control | 51 | 5.7 | 5.6 | 0.5 | 8.5 | 4.9 | 6.8 | 0 |
|  | 80°C, 20s | 51 | 4.9 | 4.9 | 0.6 | 11.8 | 3.8 | 6.3 | 0 |
|  | 80°C, 30s | 51 | 4.9 | 5.0 | 0.6 | 11.7 | 3.7 | 6.1 | 0 |
|  | control | 51 | 5.6 | 5.6 | 0.5 | 8.5 | 4.7 | 6.8 | 0 |
|  | 20°C, 30s | 102 | 6.0 | 6.0 | 0.7 | 11.1 | 1.5 | 7.2 | 0 |
|  | control | 102 | 5.6 | 5.6 | 0.5 | 8.5 | 4.7 | 6.8 | 0 |

Suppl. Table 2: ANOVA-table for the fixed effect of trial (date of sampling) and trial setting on germ count for the cold air treatment

| **Bacterium** | **Source** | **DF** | **Type III SS** | **Mean Square** | **F Value** | **p Value** |
| --- | --- | --- | --- | --- | --- | --- |
| *Campylobacter* | trial | 8 | 15.7698071 | 1.9712259 | 14.41 | <.0001 |
|  | trial setting | 6 | 134.4834979 | 22.4139163 | 163.88 | <.0001 |
| *E. coli* | trial | 8 | 79.5287315 | 9.9410914 | 17.64 | <.0001 |
|  | trial setting | 6 | 97.9802077 | 16.3300346 | 28.97 | <.0001 |
| *Salmonella* | trial | 8 | 32.8258592 | 4.1032324 | 35.78 | <.0001 |
|  | trial setting | 6 | 116.0506619 | 19.3417770 | 168.67 | <.0001 |
| TCC | trial | 8 | 33.3042479 | 4.1630310 | 18.05 | <.0001 |
|  | trial setting | 6 | 114.9928940 | 19.1654823 | 83.10 | <.0001 |

Suppl. Table 3: Results for pairwise comparison of the different trial settings for the hot water treatment using Tukey-Kramer test in post hoc analysis

| **Bacterium** | **Trial**  **setting** | **20°C, 30s** | **70°C, 30s** | **75°C, 20s** | **75°C, 30s** | **80°C, 20s** | **80°C, 30s** | **control** |
| --- | --- | --- | --- | --- | --- | --- | --- | --- |
| *Campylobacter* | 20°C, 30s | . | <.0001 | <.0001 | <.0001 | <.0001 | <.0001 | <.0001 |
|  | 70°C, 30s | <.0001 | . | 0.9933 | 0.9827 | 0.0193 | 0.9886 | <.0001 |
|  | 75°C, 20s | <.0001 | 0.9933 | . | 1.0000 | 0.0002 | 0.6720 | <.0001 |
|  | 75°C, 30s | <.0001 | 0.9827 | 1.0000 | . | 0.0001 | 0.5708 | <.0001 |
|  | 80°C, 20s | <.0001 | 0.0193 | 0.0002 | 0.0001 | . | 0.0110 | <.0001 |
|  | 80°C, 30s | <.0001 | 0.9886 | 0.6720 | 0.5708 | 0.0110 | . | <.0001 |
|  | control | <.0001 | <.0001 | <.0001 | <.0001 | <.0001 | <.0001 | . |
| *E. coli* | 20°C, 30s | . | <.0001 | <.0001 | <.0001 | <.0001 | <.0001 | <.0001 |
|  | 70°C, 30s | <.0001 | . | 0.9976 | 0.9941 | 0.9825 | 1.0000 | 0.2650 |
|  | 75°C, 20s | <.0001 | 0.9976 | . | 1.0000 | 1.0000 | 0.9855 | 0.6948 |
|  | 75°C, 30s | <.0001 | 0.9941 | 1.0000 | . | 1.0000 | 0.9717 | 0.7824 |
|  | 80°C, 20s | <.0001 | 0.9825 | 1.0000 | 1.0000 | . | 0.8438 | 0.8892 |
|  | 80°C, 30s | <.0001 | 1.0000 | 0.9855 | 0.9717 | 0.8438 | . | 0.1220 |
|  | control | <.0001 | 0.2650 | 0.6948 | 0.7824 | 0.8892 | 0.1220 | . |
| *Salmonella* | 20°C, 30s | . | <.0001 | <.0001 | <.0001 | <.0001 | <.0001 | 0.4890 |
|  | 70°C, 30s | <.0001 | . | 0.5599 | 0.4182 | 0.0130 | 0.1161 | <.0001 |
|  | 75°C, 20s | <.0001 | 0.5599 | . | 0.9999 | 0.5479 | 0.9624 | <.0001 |
|  | 75°C, 30s | <.0001 | 0.4182 | 0.9999 | . | 0.7060 | 0.9913 | <.0001 |
|  | 80°C, 20s | <.0001 | 0.0130 | 0.5479 | 0.7060 | . | 0.9466 | <.0001 |
|  | 80°C, 30s | <.0001 | 0.1161 | 0.9624 | 0.9913 | 0.9466 | . | <.0001 |
|  | control | 0.4890 | <.0001 | <.0001 | <.0001 | <.0001 | <.0001 | . |
| TCC | 20°C, 30s | . | <.0001 | <.0001 | <.0001 | <.0001 | <.0001 | <.0001 |
|  | 70°C, 30s | <.0001 | . | 0.4497 | 0.4901 | 0.9237 | 0.9882 | <.0001 |
|  | 75°C, 20s | <.0001 | 0.4497 | . | 1.0000 | 0.9673 | 0.8404 | <.0001 |
|  | 75°C, 30s | <.0001 | 0.4901 | 1.0000 | . | 0.9777 | 0.8714 | <.0001 |
|  | 80°C, 20s | <.0001 | 0.9237 | 0.9673 | 0.9777 | . | 0.9991 | <.0001 |
|  | 80°C, 30s | <.0001 | 0.9882 | 0.8404 | 0.8714 | 0.9991 | . | <.0001 |
|  | control | <.0001 | <.0001 | <.0001 | <.0001 | <.0001 | <.0001 | . |

Suppl. Table 4: Descriptive statistics per trial setting for the cold air treatment

| **Bacterium** | **Trial setting** | **N** | **Mean** | **Median** | **SD** | **CV** | **Min.** | **Max.** | **N Missing** |
| --- | --- | --- | --- | --- | --- | --- | --- | --- | --- |
| *Campylobacter* | -80°C, 20s | 51 | 2.1 | 2.0 | 0.5 | 23.1 | 1.0 | 3.7 | 0 |
|  | -80°C, 30s | 50 | 1.7 | 1.8 | 0.6 | 35.2 | 0.5 | 3.2 | 1 |
|  | -80°C, 40s | 49 | 1.8 | 1.8 | 0.6 | 31.4 | 0.3 | 3.1 | 2 |
|  | control | 51 | 1.9 | 2.0 | 0.7 | 35.3 | 0.3 | 3.7 | 0 |
|  | -90°C, 20s | 51 | 1.9 | 1.8 | 0.5 | 27.5 | 1.1 | 3.2 | 0 |
|  | -90°C, 30s | 51 | 1.8 | 1.8 | 0.5 | 29.1 | 0.6 | 3.5 | 0 |
|  | -90°C, 40s | 34 | 1.8 | 1.7 | 0.5 | 28.5 | 1.0 | 3.4 | 0 |
|  | control | 51 | 2.2 | 1.9 | 0.8 | 36.7 | 1.1 | 4.7 | 0 |
| *E. coli* | -80°C, 20s | 51 | 1.9 | 2.0 | 0.7 | 38.3 | 0.5 | 3.6 | 0 |
|  | -80°C, 30s | 50 | 1.9 | 1.9 | 0.7 | 37.4 | 0.8 | 4.5 | 1 |
|  | -80°C, 40s | 50 | 1.8 | 1.8 | 0.7 | 37.8 | 0.6 | 3.8 | 1 |
|  | control | 51 | 1.9 | 1.9 | 0.7 | 35.7 | 0.3 | 3.5 | 0 |
|  | -90°C, 20s | 51 | 2.0 | 1.9 | 0.5 | 27.2 | 1.1 | 3.3 | 0 |
|  | -90°C, 30s | 51 | 2.3 | 2.2 | 0.5 | 21.2 | 1.3 | 3.2 | 0 |
|  | -90°C, 40s | 34 | 1.7 | 1.7 | 0.4 | 24.5 | 0.9 | 2.7 | 0 |
|  | control | 51 | 2.4 | 2.3 | 0.7 | 27.4 | 1.5 | 3.8 | 0 |
| TCC | -80°C, 20s | 51 | 4.2 | 4.2 | 0.3 | 6.5 | 3.7 | 4.9 | 0 |
|  | -80°C, 30s | 51 | 4.3 | 4.2 | 0.3 | 7.5 | 3.7 | 5.0 | 0 |
|  | -80°C, 40s | 50 | 4.2 | 4.2 | 0.3 | 6.9 | 3.8 | 5.0 | 1 |
|  | control | 51 | 4.3 | 4.3 | 0.3 | 7.8 | 3.7 | 5.2 | 0 |
|  | -90°C, 20s | 51 | 4.2 | 4.2 | 0.3 | 7.5 | 3.6 | 5.3 | 0 |
|  | -90°C, 30s | 51 | 4.2 | 4.1 | 0.4 | 8.7 | 3.7 | 5.5 | 0 |
|  | -90°C, 40s | 34 | 4.3 | 4.3 | 0.4 | 8.9 | 3.5 | 5.3 | 0 |
|  | control | 51 | 4.3 | 4.3 | 0.4 | 8.5 | 3.7 | 5.8 | 0 |

Suppl. Table 5: ANOVA-table for the fixed effect of trial (date of sampling) and trial setting on germ count for the cold air treatment

| **Bacterium** | **Source** | **DF** | **Type III SS** | **Mean Square** | **F Value** | **p Value** |
| --- | --- | --- | --- | --- | --- | --- |
| *Campylobacter* | trial | 5 | 41.11617812 | 8.22323562 | 30.70 | <.0001 |
|  | trial setting | 6 | 11.14739958 | 1.85789993 | 6.94 | <.0001 |
| *E. coli* | trial | 5 | 48.81211493 | 9.76242299 | 32.30 | <.0001 |
|  | trial setting | 6 | 14.78471015 | 2.46411836 | 8.15 | <.0001 |
| TCC | trial | 5 | 1.59068016 | 0.31813603 | 2.99 | 0.0117 |
|  | trial setting | 6 | 0.88613444 | 0.14768907 | 1.39 | 0.2186 |

Suppl. Table 6: Results for pairwise comparison of the different trial settings for the cold air treatment using Tukey-Kramer test in post hoc analysis

| **Bacterium** | **Trial**  **setting** | **-80°C, 20s** | **-80°C, 30s** | **-80°C, 40s** | **-90°C, 20s** | **-90°C, 30s** | **-90°C, 40s** | **control** |
| --- | --- | --- | --- | --- | --- | --- | --- | --- |
| *Campylobacter* | -80°C, 20s | . | 0.0141 | 0.0380 | 0.0120 | 0.0020 | 0.0005 | 0.8240 |
|  | -80°C, 30s | 0.0141 | . | 0.9999 | 0.9485 | 0.7353 | 0.4027 | 0.3994 |
|  | -80°C, 40s | 0.0380 | 0.9999 | . | 0.8744 | 0.5887 | 0.2818 | 0.6106 |
|  | -90°C, 20s | 0.0120 | 0.9485 | 0.8744 | . | 0.9927 | 0.7889 | 0.0097 |
|  | -90°C, 30s | 0.0020 | 0.7353 | 0.5887 | 0.9927 | . | 0.9829 | 0.0007 |
|  | -90°C, 40s | 0.0005 | 0.4027 | 0.2818 | 0.7889 | 0.9829 | . | 0.0002 |
|  | control | 0.8240 | 0.3994 | 0.6106 | 0.0097 | 0.0007 | 0.0002 | . |
| *E. coli* | -80°C, 20s | . | 0.9994 | 0.8816 | 0.0388 | 0.9205 | <.0001 | 1.0000 |
|  | -80°C, 30s | 0.9994 | . | 0.9855 | 0.0911 | 0.9836 | <.0001 | 1.0000 |
|  | -80°C, 40s | 0.8816 | 0.9855 | . | 0.3136 | 1.0000 | 0.0009 | 0.9603 |
|  | -90°C, 20s | 0.0388 | 0.0911 | 0.3136 | . | 0.0946 | 0.0784 | 0.0012 |
|  | -90°C, 30s | 0.9205 | 0.9836 | 1.0000 | 0.0946 | . | <.0001 | 0.8394 |
|  | -90°C, 40s | <.0001 | <.0001 | 0.0009 | 0.0784 | <.0001 | . | <.0001 |
|  | control | 1.0000 | 1.0000 | 0.9603 | 0.0012 | 0.8394 | <.0001 | . |
| TCC | -80°C, 20s | . | 1.0000 | 0.9999 | 0.8341 | 0.9744 | 1.0000 | 0.9993 |
|  | -80°C, 30s | 1.0000 | . | 0.9989 | 0.7702 | 0.9523 | 1.0000 | 1.0000 |
|  | -80°C, 40s | 0.9999 | 0.9989 | . | 0.9267 | 0.9948 | 0.9989 | 0.9867 |
|  | -90°C, 20s | 0.8341 | 0.7702 | 0.9267 | . | 0.9956 | 0.4059 | 0.2234 |
|  | -90°C, 30s | 0.9744 | 0.9523 | 0.9948 | 0.9956 | . | 0.7653 | 0.6076 |
|  | -90°C, 40s | 1.0000 | 1.0000 | 0.9989 | 0.4059 | 0.7653 | . | 1.0000 |
|  | control | 0.9993 | 1.0000 | 0.9867 | 0.2234 | 0.6076 | 1.0000 | . |
